# Tuning Hydrogen versus Methane Production on Sustainable Biochar-Based Cathodes in Microbial Electrolysis Cells by Voltage Control

**DOI:** 10.1021/acsomega.6c00714

**Published:** 2026-03-05

**Authors:** Gabriele Soggia, Andrea Goglio, Elisa Clagnan, Tommy Pepè Sciarria, Barbara Mecheri, Alessandra D’Epifanio, Jillian L. Goldfarb, Piergiorgio Stevanato, Pierangela Cristiani, Fabrizio Adani

**Affiliations:** a Gruppo Ricicla Lab., Department of Agricultural and Environmental Science, 9304 University of Milan, Via Celoria 2, Milan 20133, Italy; b Department of Chemical Science and Technologies, 9318 University of Rome Tor Vergata, Via della Ricerca Scientifica, Rome 00133, Italy; c Smith School of Chemical and Biomolecular Engineering, Cornell University, Ithaca, New York 14853, United States; d Department of Agronomy, Food, Natural Resources, Animals and Environment (DAFNAE), University of Padova, Viale dell’Università 16, Legnaro 35020, Italy; e RSE - Ricerca sul Sistema Energetico S.p.A., Via Rubattino 54, Milano 20134, Italy

## Abstract

Due to the intermittency of solar and wind energy generation, efficient energy storage solutions are essential to ensure a global transition to renewable energy sources. Bioelectrochemical Power-to-Hydrogen systems are a promising storage pathway, yet their development is limited by high costs and low productivity compared to conventional hydrogen production. Novel, sustainable, and cost-effective materials, such as carbon-based electrodes, can help to overcome these challenges. This study evaluates five cathodes for hydrogen and methane production in microbial electrolysis cells (MECs) operated at 600 and 800 mV: stainless steel mesh (SSM), two custom-made biochars derived from olive mill waste (OMW-1, OMW-2), and two commercial carbon-based materials (Carbon Black and Black Pearls). OMW-1 achieved a H_2_ yield of 257 ± 62 mL L^–1^ d^–1^ at 800 mV, showing the potential of noncommercial biochar. CB and SSM performed better, reaching 493 ± 57 and 496 ± 9 mL L^–1^ d^–1^ H_2_, respectively. Cyclic voltammetry and next-generation sequencing revealed that hydrogen-oxidizing bacteria colonization negatively impacted H_2_ yields. At 600 mV, increased CH_4_ production was observed for OMW-2, BP, and CB. Energetically, OMW-2 (3.0 ± 0.2 kWh L^–1^ d^–1^) performed comparably to CB and BP (both 3.3 kWh L^–1^ d^–1^), outperforming SSM at both voltages. These findings support the viability of carbon-based cathodes as sustainable alternatives to metal-based ones with the potential to reduce electrode costs while maintaining or improving energy productivity.

## Graphical abstract

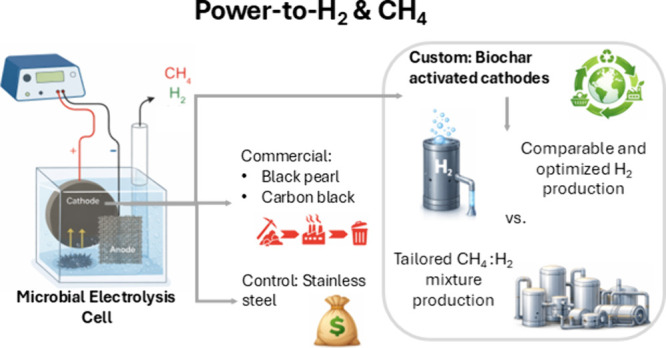

## 1 Introduction

Embracing low- or zero-carbon electricity production systems is crucial to mitigate global warming and climate change in the immediate future.^1^ Renewables are expected to account for 46% of the global energy mix by 2030.^2^ A significant portion of renewable electricity will be produced from intermittent sources like solar and wind that are forecasted to be the dominant contributors to renewable energy production, accounting for 15% of the global electricity generation that corresponds approximately to half of the total renewable share (32% of the global electricity generation).^2^

Intermittent renewable energy sources are nonprogrammable as they are influenced by diurnal cycles, climatic conditions, and seasonality that can reduce their production down to zero.^3^ To ensure a stable energy supply, surplus electricity generated during peak production periods must be stored.^4^ Different energy storage technologies at various stages of development (i.e., Technology Readiness Levels) are available. These systems are categorized based on energy conversion type: mechanical (e.g., flywheel, compressed air, pumped hydro), electrochemical (e.g., batteries and supercapacitors), bioelectrochemical (e.g., bioelectrochemical Power-to-X approach), thermal, and chemical.^5^

Among storage technologies, Power-to-Gas (PtG) stands out as a promising solution, allowing the conversion of renewable electricity into a storable and transportable gas carrier, such as methane (Power-to-Methane, PtM) and hydrogen (Power-to-Hydrogen, PtH). These gases can then serve as sustainable fuels and, particularly hydrogen (H_2_), as feedstock to produce chemicals such as methane (CH_4_), ammonia, and methanol.^3^ H_2_ has a higher specific energy than methane, i.e., ∼120 MJ kg^–1^ versus 50 MJ kg^–1^, and a higher energy density compared to other hydrocarbons, therefore making H_2_ a good candidate for energy storage and large-scale and long-distance transportation.^6^

In 2023, the global H_2_ demand reached 97 Mt; it was primarily met through production from unabated fossil fuels such as steam methane reforming and oil or coal gasification.^7^ Decoupling H_2_ production from fossil fuel is crucial to make H_2_ a net zero fuel. The production of green H_2_ from renewable energy is gaining momentum due to advancements in electrolysis technologies.^7,8^ The adoption of electrolysis technologies, however, faces several challenges, including high costs of membranes and catalysts, significant electricity requirements, and the need for high-purity water.^9^ To address these limitations, bioelectrochemical systems (BES), particularly microbial electrolysis cells (MECs), have emerged as promising alternatives to conventional electrolysis.

MECs enable high-purity H_2_ production with high yields and energy efficiency.^10^ The adoption of a biocathode functionalized with carbon materials, e.g., biochar, can help reduce the cost and environmental impact of H_2_ production as compared to electrolysis technology. Electrolysis requires noble metal catalysts (e.g., platinum, palladium, and gold), which are easily contaminated and inactivated, leading to increased overpotentials and low H_2_ production.^10^ New research is needed to develop, characterize, and select novel, sustainable, and effective electrode materials. Biochar- and carbon-derived cathodes are particularly interesting due to their desirable properties of stability, high conductivity, large surface area, biocompatibility, and lower cost compared to metal-based and other carbon-based catalysts.^11^ For example, the cost of biochar can range from 51 to 381 USD ton^–1^,^12^ while graphite-based materials can reach costs up to 2500 USD ton^–1^. Furthermore, biochar is considered a renewable catalyst due to the nature of starting materials, usually waste, making it more sustainable compared to nonrenewable metal- and commercially available carbon-based materials.^13^

Additionally, MECs leverage biological anodic oxidation, allowing for simultaneous H_2_ production and organic waste or wastewater treatment.^10^ Within MECs, anodic electroactive bacteria (EAB), specifically exoelectrogenic microorganisms, thrive on the anode surface. EAB oxidize organic matter (eq 1) and produce H^+^ using the anode as the terminal electron acceptor of their electron transport chain through peculiar structure such as nanowires, cytochromes, or other proteins.^14,15^ Electrons move to the cathode through an external circuit where H^+^ is reduced to H_2_ (eq 2).^9,15^ A small external voltage is needed to overcome the thermodynamical barrier and drive H_2_ synthesis at the cathode.^16^ The minimum theoretical cell potential difference (*U*) to be applied is calculated as the difference between cathode and anode redox potentials, that is, at pH 7, 0.13 V (eq 3). However, due to overpotentials, i.e., activation, Ohmic, and concentration overpotentials, the required cell potential difference is generally higher, between 0.5 and 1.1 V.^17^

$${\mathrm{CH}}_{3}\mathrm{COOH}+2H_{2}O\to 2{\mathrm{CO}}_{2}+8H^{+}+8e^{-}\;E_{0}^{\prime }=-0.28\;V\;\mathrm{vs}\;\mathrm{SHE}$$ 1

$$2H^{+}+2e^{-}\to H_{2}\;E_{0}^{\prime }=-0.41\;V\;\mathrm{vs}\;\mathrm{SHE}$$ 2

$${\mathrm{CH}}_{3}\mathrm{COOH}+2H_{2}O\to 2{\mathrm{CO}}_{2}+4H_{2}\;U=-0.13\;V$$ 3

MEC performance can be hindered by competing reactions that divert electrons away from H_2_ production, contaminating the produced gas. For example, methanogenic Archaea can produce CH_4_ via acetoclastic metabolism by consuming volatile fatty acids (eq 4) or hydrogenotrophic metabolism by using H_2_ as an electron donor (eq 5).^17^

$${\mathrm{CH}}_{3}\mathrm{CO}O^{-}+H_{2}O\to {\mathrm{CH}}_{4}+\mathrm{HC}{O_{3}}^{-}+8e^{-}\;E_{0}^{\prime }=-0.29\;V\;vs\;SHE,\;pH\;7$$ 4

$$4H_{2}+\mathrm{HC}{O_{3}}^{-}+H^{+}\to {\mathrm{CH}}_{4}+3H_{2}O\;E_{0}^{\prime }=-0.24\;V\;\mathrm{vs}\;\mathrm{SHE},\;\mathrm{pH}\;7$$ 5

For example, Hou et al.^18^ observed a negligible concentration of CH_4_ at the beginning of the experiment, with most of the produced gas being H_2_ (95 ± 0.8%). However, after 10 cycles, CH_4_ reached 30 ± 5.4%, while H_2_ dropped to 70 ± 9.2%. Similarly, Zhang et al.^19^ reported a decline in H_2_ production from 6.59 ± 0.2 mol mol^–1^ glucose to 0.43 ± 0.05 mol mol^–1^ glucose in favor of CH_4_ that passed from 0.15 ± 0.07 mol mol^–1^ glucose to 1.76 ± 0.1 mol mol^–1^ glucose in a MEC system operated at 0.5 V with 1 g L^–1^ glucose as the substrate; overall hydrogen recovery decreased from 61 ± 4% to 18 ± 1%. For these reasons, there is a need to find effective approaches for methanogenic activity limitation to increase H_2_ yield and purity. To tackle the environmental impact and MEC cost, the first aim of this study was to evaluate the performance of five different electrodes for H_2_ production in an MEC configuration. One electrode was made of commercial stainless-steel mesh, two were made from carbon cloth functionalized with commercial carbon-based materials, and two from carbon cloth functionalized with olive mill waste biochar having different physical and chemical characteristics due to treatment applied before and after pyrolysis. To address the CH_4_ contamination challenge, the second aim was to assess the impact of two different cell potential differences (800 and 600 mV) on H_2_ and CH_4_ production and finally to analyze how variations in cell polarization influence gas yields and microbial communities. To the best of the authors' knowledge, no MEC studies were found in the literature using noncommercial biochar-functionalized carbon cloth with similar characteristics, as carbon cloth cathodes are typically doped with a metallic catalyst such as platinum, nickel, cobalt, and molybdenum.^20^

## 2 Materials and Methods

Five different electrodes were prepared and tested in single-chamber MECs. The biofilm grown on the anode bristles before and after electrochemical testing was sequenced to understand the interplay between cell polarization, electrode, and microbial communities present.

### 2.1 Cathodes

Cathodes were prepared from three commercial materials, and two biochars were fabricated in-house.

#### 2.1.1 Biochar Preparation

The selection of the olive mill waste (OMW) biochar was based on the findings of Pepè Sciarria et al.^21^ that compared the performance of three different OMW-based biochars using the microbial fuel cell (MFC) and electrochemical techniques. The two biochars with the highest catalytic activity, measured by reaction rate, active site density, and electron exchange, were selected for this study. Their superior performance was attributed to a higher electrode double-layer capacitance, which enhanced the exposure of the effective active sites.

Olive mill waste (OMW) was collected from an olive oil production plant in Calabria, Italy. Two biochars were produced from OMW with and without pretreatment according to Goldfarb et al.^22^ Briefly, the pretreatment involved supercritical pure CO_2_ extraction at a total pressure of 250 bar and a temperature of 70 °C, with a CO_2_ flow rate of 80 kg h^–1^. The pretreatment step was intended to recover bioactive compounds such as polyphenols and polyunsaturated fatty acids from the OMW and develop a porous structure to increase the surface area of the resulting biochar. Supercritical CO_2_ extraction causes structural changes and has significant effects on surface functional groups. Both pretreated and non-pretreated OMWs were then pyrolyzed at 600 °C for 30 min under a high purity nitrogen flow to obtain the two biochars.

The two biochars were then subjected to two different activations. Briefly, the pretreated OMW biochar was chemically activated by using potassium hydroxide (KOH), a porogen agent. The goal of activation treatments is to develop a well-structured porous network in biochar, thereby increasing its surface area (OMW-1). The non-pretreated OMW biochar underwent physical activation with high purity CO_2_ at a flow rate of 100 mL min^–1^ at 800 °C for 45 min (OMW-2). These treatments also enhance the presence of oxygen-containing functional groups. Both chemical (KOH) and physical (CO_2_) activation methods lead to an increase in hydroxyl (−OH) groups compared to inactivated biochar with differences in terms of relative abundance of −OH and carbonyl (−C=O) groups.^22^

#### 2.1.2 Cathode Preparation

Five different circular electrodes (3.8 cm diameter; 11.3 cm^2^ surface) were tested for hydrogen production by using MEC (Figure 1). Four out of five were produced using carbon cloth (ELAT LT1400W MPL treated, FuelCellsEtc, USA) as a conductive support by incorporating a gas diffusion layer composed of (i) the pretreated OMW biochar (OMW-1, see Section 2.1.1), (ii) the non-pretreated OMW biochar (OMW-2, see Section 2.1.1), (iii) Carbon Black (CB, Vulcan XC72R, Cabot Corporation, MA, USA), and (iv) Black Pearls 2000 (BP, Cabot Corporation, MA, USA). Previous works highlighted that biochar functionalization of the electrodes improved the overall performance in MFC, MEC, and microbial electrosynthesis cell (MES) compared to bare carbon cloth.^23–25^ For this reason, no bare carbon cloth control was considered. CB is a conductive material with good functionality at relatively low loading compared to other commercial products. It is easy to disperse, and it has a low sulfur content and minimal ionic contamination.^26^ BP is a very fine powder that offers a high surface area and high conductivity.^27^ The gas diffusion layer was applied by brush painting a suspension of OMW-1, OMW-2, CB, and BP following the procedure and loading specifications described in Pepè Sciarria et al.^21^ Briefly, biochar was suspended (0.185 mg mL^–1^) in a solution of 2-propanol (31 vol%), deionized water (7.5 vol %), and Nafion perfluorinated resin solution (61.5 vol %, Sigma-Aldrich, Germany). The resulting suspension was applied to the cathode by brush coating, dried at room temperature, and then pressed at 90 °C for 2 min at 2 bar, yielding a final biochar loading of 5 mg cm^–2^. In order to compare the results of carbon-based electrodes with a benchmark material, the fifth electrode consisted of a stainless-steel mesh (SSM) that has already been applied in BES and has lower cost compared to the noble metal catalyst. A summary of the cathode compositions and manufacturing procedures is provided in Table 1.

**Figure 1:**
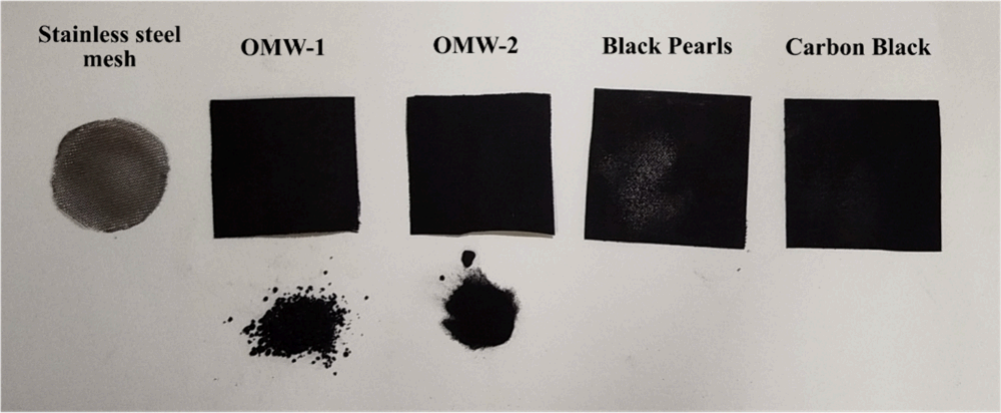
Cathodes used for H_2_ production in MEC before the experiment; the stainless steel mesh (SSM) cathode was used as reference. In the lower part of the figure, the biochar powders can be seen.

**Table 1:** Description of Compositions and Manufacturing of Experimented Cathode Materials

electrode	support	biochar	biochar activation	GDL characteristic	**biochar surface area** **(m** **^2^** **g** ^**–1**^ **)** ^a^
OMW-1	carbon cloth	OMW biochar by supercritical pure CO_2_ extraction	chemical activation with KOH		444 (250,860)
OMW-2	carbon cloth	OMW biochar	100 mL L^–1^ high purity CO_2_ (800 °C, 45 min)		658 (371,770)
CB	carbon cloth	commercial Carbon Black		trace of metals 0.5% and sulfur 2%	250 (141,250)
BP	carbon cloth	commercial Black Pearls		high purity	1,400 (791,000)
SSM	stainless steel mesh (AISI 304)				

a Surface area values for OMW biochar are from Pepè Sciarria et al.,^21^ for CB from Perez-Rodriguez et al.,^28^ and for BP from Cangül et al.^29^ In brackets, the value of surface area (cm^2^) was calculated by multiplying the cathode surface area by biochar loading and biochar surface area.

### 2.2 Bioelectrochemical Experiments

Bioelectrochemical H_2_ production was conducted in single-chamber MECs with a total volume of 28 mL using a two-electrode setup. Each MEC was equipped with the cathodes described in Section 2.1.2 and a graphite fiber brush anode with a titanium wire core (Panex 33 160 K, Zoltek, USA). The anode had a length and diameter of 2.5 cm, a surface area of 0.22 m^2^, a brush-volume of 18,200 m^2^ m^–3^, and a porosity of 95%.

To facilitate gas recovery, the MECs were modified with a 16 mL glass pipe as described in Goglio et al.^23^ and connected to a gas bag for gas collection.

Two different cell potentials (800 and 600 mV) were tested using a DC power supply (IPS 3303D, ISO-TECH DC Power Supply, Merseyside, England). These two polarizations were selected to investigate the effect of cell voltage difference on H_2_ and CH_4_ production, given that H_2_ production has a higher theoretical voltage requirement (0.13 V) compared to CH_4_ production (0.04 V). Anode and cathode potentials were monitored with a data logger (Keithley 2700 Multimeter/Data Acquisition/Switch Systems, USA). The two different polarizations were applied sequentially to observe the microbial community response to a lower cell voltage with the goal of improving energy efficiency and reducing overall operational costs. Henceforth, all electrode potentials are reported relative to the silver/silver chloride (Ag/AgCl) reference electrode.

#### 2.2.1 Experimental Procedures

The MECs were inoculated with 12.5 mL of an inoculum from a previous experiment^21^ and 12.5 mL of synthetic medium with 2 g L^–1^ acetate. The synthetic medium composition per liter was 982.5 mL of phosphate buffer solution (PBS), 12.5 mL of mineral solution, and 5 mL of vitamin solution.^23^ After an acclimation period of 7 days to adapt the microbial community to acetate as a substrate and to the electrode as the terminal electron acceptor, the MECs were operated at a controlled temperature of 28 ± 1 °C for 17 days. The medium and C source were replaced every two days; 8 cycles were performed over the 17 day experimental period.

The produced gas was quantified using a graduated syringe as indicated by Lu et al.^30^ and Gautam et al.,^31^ while the composition was analyzed by means of a 990 Micro GC System (Agilent, Santa Clara, USA).

#### 2.2.2 Calculations

The productivity or space-time yield (STY) was determined as cumulative H_2_ (*V*_H_2__, mL) divided by the MEC volume (*V*_MEC_, 28 × 10^–3^ L) and experiment time length (*t*, 17 days), as shown in eq 6:

$$\mathrm{STY}\;(mL\;L^{-1}\;d^{-1})=\frac{V_{H_{2}}}{V_{\mathrm{MEC}}\times t}$$ 6

A 17 day period was used to obtain an average hydrogen production rate, providing a more representative and reliable assessment of long-term reactor productivity.

Space-time yield (STY) was preferred, as terminology, over hydrogen production to explicitly underline the normalization of hydrogen generation by both time and reactor volume. Commonly used in works addressing reactor performance, biotechnological and industrial process evaluation, and scalability, the use of STY also at this lab-scale could supply data comparison for further scale up.

Coulombic efficiency (CE) was calculated as described in eq 7:

$$\mathrm{CE}\;(\%)=\frac{V_{H_{2}}}{{\mathrm{mol}}_{{\mathrm{CH}}_{3}\mathrm{COOH}}\times n\times {\mathrm{MW}}_{H_{2}}\times \mathrm{MV}}$$ 7

Conventional MEC metrics (e.g., Coulombic efficiency, STY, etc.) describe the electrochemical efficiency or reactor productivity but do not directly express the energetic value of the produced hydrogen. Total theoretical energy was calculated as a complementary indicator to provide an evaluation of the cumulative energy potential of hydrogen production, facilitating the evaluation of the overall energy recovery potential of the system. The total theoretical energy from the produced gas mixture was calculated as the sum of the theoretical energy gain (*E*) calculated for each single components of the gas mixture (H_2_ and CH_4_) as described in eq 8:

$$E\;(\mathrm{kWh})=\frac{V}{V_{m}}\times \mathrm{MW}\times \mathrm{HV}$$ 8

where *V* is the volume of the gas produced (L), *V*_m_ is the molar volume at 28 °C and 1 atm pressure (equal to 24.69 L mol^–1^), MW is the molecular weight (2.016 g mol^–1^ for H_2_ and 16.04 g mol^–1^ for CH_4_), and HV is the heating value (also known as calorific value or specific energy) (120 kJ g^–1^ for H_2_ ^31^ and 50 kJ g^–1^ for CH_4_ ^32^). Energy productivity (kWh L^–1^ d^–1^) was obtained by dividing the total theoretical energy by the volume of the bioreactor and days of experiment.

#### 2.2.3 Electrochemical Analysis

Cyclic voltammetry curves were recorded at the end of both 600 and 800 mV experimentations using a potentiostat (2551, AMEL, Italy) and a 3 M Ag/AgCl as the reference electrode in a standard three-electrode setup. The potential range was between −0.8 V and +0.8 V vs Ag/AgCl at a scan rate of 10 mV s^–1^.

### 2.3 16S rRNA Multi-Amplicon Sequencing

DNA extraction was performed from (i) the MEC electrolyte and the biofilm grown on the anode bristles after the acclimation, before the start of the H_2_ production experiment, and (ii) the biofilms grown on the anode bristles at the end of the two experimental phases at different cell voltages (800 and 600 mV). The microbial community grown on the cathode was sampled only after the 600 mV polarization phase to avoid potential cathode damage, as the 600 mV polarization had been applied immediately after 800 mV voltage application, as described in Section 2.2.

DNA was extracted using the DNeasy PowerSoil Kit (Qiagen, Germany) according to manufacturer instructions after an initial step of thermal treatment (5 cycles of 10 min at −20 °C and 10 min at 65 °C). The yield and purity (*A*_260_/*A*_280_ and *A*_260_/*A*_230_) of the extracted DNA were quantified on a Nanodrop 1000 spectrophotometer (Thermo Fisher Scientific), while eventual fragmentation was determined through gel electrophoresis 0.8% (w/v) 1× TAE agarose gels. DNA was stored at −80 °C until analysis.

Multi-amplicon sequencing and bioinformatics were performed as per Maretto et al.^33^ The nucleotide sequences generated and analyzed are available at the NCBI SRA repository (BioProject accession number: PRJNA1244633).

All statistical analyses were performed in R Studio (version 4.4.2). Taxonomic summaries were performed using the phyloseq package.^34^ Observed richness, Shannon diversity, and Pielou’s evenness indexes were calculated using the package "vegan".^35^ Following the Shapiro–Wilk test to test normality, differences among samples of normally distributed data were tested by one-way analysis of variance (ANOVA), followed by Tukey’s post hoc test (*p* < 0.05), while not normal data were analyzed through a nonparametric Kruskal–Wallis test, followed by Dunn’s test for multiple comparisons. For pairwise comparison, the *t* test and Wilcoxon signed rank test were used for not mal and not normal data, respectively.

Multivariate analyses were performed on OTU relative abundances. To test the effect of the materials (i.e., OMW-1, OMW-2, BP, CB, and SSM) and of the polarizations (i.e., 800 and 600 mV), first, nonmetric multidimensional scaling (NMDS) based on Bray–Curtis distances was applied, and then, results were confirmed through a PERMANOVA test (vegan package). The betadisper function (vegan package) was further used to understand the variance followed by the simper function to understand the main differences in the OTU composition. Venn diagrams were drawn using the web tool http://bioinformatics.psb.ugent.be/webtools/Venn/, while enzyme profiles for specific pathways were investigated through iVikodak.^36^

### 2.4 Statistical Analysis

All experiments and analyses were performed using three replicates. Mean and standard deviation (SD) values were determined via R Studio. Determination of significant differences among the parameters analyzed at a level of significance of *p* < 0.05 was carried out by analysis of variance (ANOVA) and Tukey’s post hoc test using R Studio.

## 3 Results and Discussion

### 3.1 Microbial Electrolysis Cell Performance

The performance of the MECs was evaluated by measuring the H_2_ production. CH_4_ concentration was monitored to assess the possible occurrence of competing reactions that could lead to acetate consumption at the anode (e.g., methanogenesis via the acetoclastic pathway) or electron withdrawal at the cathode (e.g., methanogenesis through direct electron transfer; hydrogenotrophic mechanism).^37^ The H_2_ and CH_4_ space-time yields (STYs) for each electrode are reported in Table 2. H_2_ and CH_4_ cumulative production curves are reported in Figure 2.

**Figure 2:**
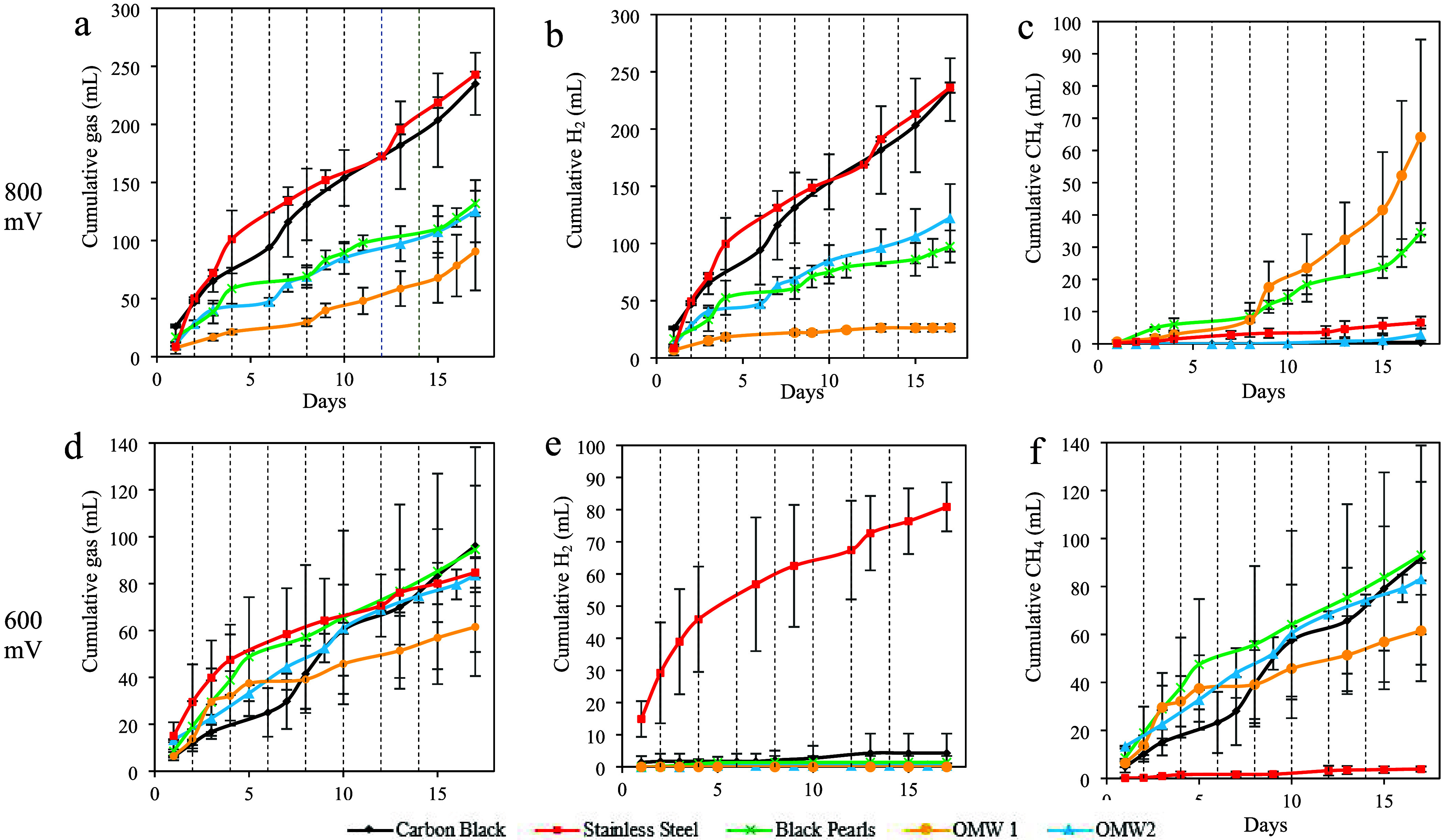
Gas production. (a) Cumulative gas production (mL) at 800 mV; (b) cumulative H_2_ production (mL) at 800 mV; (c) cumulative CH_4_ production (mL) at 800 mV; (d) cumulative gas production (mL) at 600 mV; (e) cumulative H_2_ production (mL) at 600 mV; (f) cumulative CH_4_ production (mL) at 600 mV. Error bars indicate standard deviation (*n* = 3). Dotted lines indicate the cycles.

**Table 2:** Space-Time Yields (STYs) of H_2_ and CH_4_ Expressed in Terms of mL L^–1^ d^–1^ ^a^

electrode	**voltage** (mV)	**H** **_2_** (mL L^–1^ d^–1^)	**CH** **_4_** (mL L^–1^ d^–1^)	**cumulative STY** (mL L^–1^ d^–1^)	**cumulative production** (mL)	**total energy** (Wh)	**energy productivity** (kWh L^–1^ d^–1^)	**Coulombic efficiency** (%)
OMW-1	600	n.d.	129 ± 44ab	129 ± 44a	62 ± 21a	1.3 ± 0.5a	2.2 ± 0.7a	n.d.
	800	55 ± 7ab	135 ± 64ab	190 ± 70a	91 ± 34a	1.5 ± 0.7a	2.4 ± 1.1a	7 ± 1a
OMW-2	600	1a^b^	175 ± 14ab	176 ± 15a	84 ± 7a	1.8 ± 0.1a	3.0 ± 0.2a	0.2a^b^
	800	257 ± 62c	6 ± 6a	263 ± 56a	125 ± 27a	0.4 ± 0.0 a	0.6 ± 0.0a	33 ± 8b
CB	600	9a^b^	193 ± 67b	202 ± 54a	96 ± 26a	2.0 ± 0.7a	3.3 ± 1.1a	2a^b^
	800	493 ± 57d	1a^b^	493 ± 56b	235 ± 27b	0.6 ± 0.1a	1.1 ± 0.1a	62 ± 7c
BP	600	3a^b^	196 ± 96b	199 ± 92a	95 ± 44a	2.0 ± 1.0a	3.3 ± 1.6a	1a^b^
	800	205 ± 29c	72 ± 6ab	277 ± 23a	132 ± 11a	0.4 ± 0.0a	0.6 ± 0.0a	26 ± 4b
SSM	600	169.9 ± 16bc	8 ± 2a	178 ± 14 a	85 ± 7a	0.3 ± 0.0a	0.5 ± 0.0a	22 ± 2b
	800	496 ± 9d	14 ± 4a	510.1 ± 3b	243 ± 3b	0.8 ± 0.0a	1.3 ± 0.0a	63 ± 1c

a The reported values are expressed as mean ± standard deviation (*n* = 3). Letters following means (for each column) indicate statistically significant differences (*p* < 0.05; ANOVA, Tukey test).

b St. Dev. not shown since two replicates out of three had zero production.

Considering the cumulative gas (H_2_ + CH_4_) production at 800 mV (Table 2), SSM reached 243 ± 3 mL after 17 days, followed by CB with 235 ± 27 mL. The other cumulative production, namely, BP with 132 ± 11 mL, OMW-2 with 125 ± 27 mL, and OMW-1 with 91 ± 34 mL, were not statistically different. At 600 mV, the cumulative gas produced was not statistically different with values of 96 ± 26 mL for CB, 95 ± 44 mL for BP, 91 ± 34 mL for OMW-1, 85 ± 7 mL for SSM, and 62 ± 21 mL for OMW-2.

Considering H_2_ production at an 800 mV voltage, SSM and CB cathodes outperformed the others, achieving STYs of 496 ± 9 mL L^–1^ d^–1^ and 493 ± 57 mL L^–1^ d^–1^, respectively. The better performance of the SSM cathode can be attributed to the presence of some elements in the alloy, such as nickel and iron, which can serve as catalytic sites.^38^ The H_2_ productivity observed with the SSM cathode aligned with the values reported in the literature for the stainless steel-based cathode, which range at 0.9 V from 350 ± 80 mL L^–1^ d^–1^ to 1500 ± 40 mL L^–1^ d^–1^ at 0.9 V.^39^ In a study by Son and colleagues, similar conditions to the one used in this study were adopted, i.e., single-chamber MEC, carbon brush anode, SSM cathode, 2 g L^–1^ acetate, and 0.9 V cell voltage, resulting in similar H_2_ productivity values, i.e., 0.57 L L^–1^ d^–1^.^40^ Similarly, Kim and Logan reported a hydrogen production rate of 0.38 ± 0.04 L L^–1^ d^–1^ using an SS cathode loaded with activated carbon and Ni powder.^41^ However, the authors stated that the lower results compared to literature could be due to different types of MEC configurations (two chambers with an anion exchange membrane) and type of medium (synthetic fermentation effluent, acetate 0.27 g L^–1^). In any case, especially for the next studies, the mesh type (wire diameter, pore size, geometry of the mesh, and percent open area) should be kept into account because it can influence the results in terms of H_2_ evolution rate, cathodic H_2_ recovery, and Coulombic efficiency.^42^ The similar CB performance was attributed to its chemical and physical properties such as conductivity and surface area (in the order of 250 m^2^ g^–1^).^28^ In a study by Fujinawa and co-workers, the addition of CB to the MEC cathode chamber led to higher amount of H_2_ produced compared to the control MEC without CB, and inhibition of methanogenesis was observed, too.^43^ The OMW-2 and BP cathodes produced less than the above-discussed cathode, i.e., STYs of 257 ± 62 and 205 ± 29 mL L^–1^ d^–1^, respectively, while the OMW-1 electrode exhibited the lowest H_2_ productivity at 800 mV (i.e., 55 ± 7 mL L^–1^ d^–1^). The BP cathode has very high surface area, 1400 m^2^ g^–1^, that, considering the biochar loading and the cathode surface, corresponded to 791,000 cm^2^.^29^ A high surface area value is likely correlated with a high electric resistance that could have reduced the cathodic H_2_ production favoring the action of acetoclastic and hydrogenotrophic methanogens.^44,45^ When the surface area is increased, if most of the porosity is represented by micropores with dimensions lower than 1 μm that are inaccessible to electroactive bacteria, pore clogging or covering by the biofilm can lead to ion transport limitations and thus an increase of overall charge resistance.^46^ On the other hand, the higher productivity of OMW-2 compared to OMW-1 can be attributed to its higher BET surface area (658 m^2^ g^–1^ vs 444 m^2^ g^–1^), which seemed to meet the optimal value of porosity and electrical resistance, and to the presence of aliphatic C–H surface functional groups, as indicated by FT-IR analysis.^21^ Using the same configuration and voltage but with biochars derived from wood chips and organic fraction of municipal solid waste, prior work obtained only approximately 50 mL of a gas mixture of H_2_ and CH_4_ after 17 days (CH_4_ and H_2_ were not considered separately).^23^ This corresponded to an STY of approximately 100 mL L^–1^ d^–1^, which is lower than the cumulative H_2_ production observed in this study with noncommercial biochar. Specifically, at 800 mV, cumulative H_2_ production reached 173 ± 15 and 148 ± 50 mL for OMW-1 and OMW-2, respectively. Similar H_2_ productivity values were observed by Gautam et al.,^31^ who obtained a STY of 172 mL L^–1^ d^–1^ after 7 days of experiment using a 1 L BES with a working volume of 0.75 L, equipped with bare carbon cloth electrodes with a cell voltage of 800 mV. The different H_2_ production could be related to the different conductivity of the electrode. SSM is known for having a high conductivity (∼1.39 × 10^4^ S cm^–1^). BP and CB have similar conductivity, i.e., 2–3 S cm^–1^.^47,28^ On the other hand, OMW-based electrodes have lower conductivity in the range of 0.3–0.4 S cm^–1^. The lower conductivity of the biochar-based electrode could explain the lower H_2_ productivity.

When looking at CH_4_ production at 800 mV, low productivity values were obtained except from the samples from OMW-1 and BP. CB STY was 1 mL L^–1^ d^–1^, OMW-2 6 ± 6 mL L^–1^ d^–1^, SSM 14 ± 4 mL L^–1^ d^–1^, BP 72 ± 6 mL L^–1^ d^–1^, and OMW-1 135 ± 64 mL L^–1^ d^–1^. At a 600 mV cell voltage, OMW-1 did not produce any H_2_. At 600 mV, no significant differences were observed among the OMW-2, BP, and CB electrodes (Table 2). The standard deviation values for OMW-2, BP, and CB cathodes are not reported since two replicates out of three produced no detectable hydrogen. The SSM cathode at 600 mV produced 170 ± 16 mL L^–1^ d^–1^ H_2_, a value comparable to those of BP and OMW-1 at the same potential.

CH_4_ production values at 600 mV were higher compared to those at 800 mV for BP, CB, and OMW-2 (Table 2). The STY values for the OMW-1 and SSM remained similar to those observed at 800 mV. These results indicate that a higher cell potential difference, i.e., 800 mV, enhances H_2_ productivity compared to 600 mV, depending on the electrode material. This observation aligns with evidence from Goren et al.,^20^ where a substantial impact of applied voltage on H_2_ production was reported by using stainless steel electrodes, with higher STYs achieved at increased cell potentials. The authors hypothesized that the lower voltage could have led to electron accumulation at the anode, reducing the electron transfer rate to the cathode.^20^ Furthermore, 600 mV cell voltage could not be enough to support the H_2_ production due to overpotentials and system internal resistance. The lower speed of H_2_ production observed at 600 mV, coupled with long cycle duration, could have favored the activity of methanogens. As reported in a recent study by Son and colleagues using the same MEC configuration and similar conditions, the methane share in the biogas produced increased when the cycle duration exceeded 24 h.^48^

Contrary to H_2_ STY, no clear trend was observed between the electrode type and the cell voltage in relation to CH_4_ production. These results suggest that the higher CH_4_ production at a lower cell voltage for some of the tested cathodes may be due to slower metabolism of electroactive bacteria, leading to acetate accumulation in the MEC electrolyte. This accumulation likely promoted CH_4_ production via acetoclastic methanogens. However, the potential contribution of hydrogenotrophic methanogenesis cannot be entirely ruled out since hydrogenotrophic methanogens have a higher growth rate than acetoclastic methanogens.^40^ In a previous study by Wang and co-workers,^49^ a correlation between lower cell voltage and increased CH_4_ production was observed at the expense of H_2_ production. In this study, a decrease in CH_4_ productivity and percentage in the produced gas was reported when the cell potential difference increased from 0.3 to 0.9 V.^49^ Furthermore, the authors suggested that reducing the cycle length could be an effective strategy to minimize CH_4_ production.^48,49^ To mitigate methanogenic activity, various strategies have been explored and they were categorized as follows: (a) physical: lowering hydraulic retention time, irradiating with ultraviolet radiation, lowering the temperature, heat or oxygen pretreatment, and system exposition to the air; (b) chemical: increasing cathode potential or cell voltage, controlling pH, pretreating the substrate; (c) metabolic: growth inhibitor addition (2-bromoethanesulfonate, 2-BES) and repression of methanogenic gene expression; (d) structural: selective catalysts and materials, selective coatings, reactor configurations, and operation (periodic cleaning or biofilm disruption).^32,40,50^ In future studies, molecular and microbial characterization of these systems could give further insights on the microbial structure of these processes and on the drivers of failure or differences in outcome. In this context, a better understanding of the microbial community could be additionally used to mitigate CH_4_ production through bioaugmentation or selective enrichment of electroactive bacteria that are able to outcompete acetoclastic methanogens at the anode and hydrogen-producing electrotrophs over hydrogenotrophic methanogens in order to support the abiotic cathodic H_2_ production. Synthetic biology approaches with metabolic/genetic modification could also have further applications and beneficial effects on H_2_ production via MECs.

Regarding the produced gas composition, a higher H_2_ percentage was observed when an 800 mV potential was applied to the MEC. SSM, CB, and OMW-2 maintained relatively stable H_2_ percentages throughout the experiment, with average values of 86 ± 1%, 82 ± 5%, and 71 ± 5%, respectively. However, for BP and OMW-1 cathodes, the H_2_ share deteriorated over time at 800 mV, decreasing from 61% to 35% (48 ± 15%) and from 65% to 0.87% (24 ± 26%), respectively. At 600 mV, only the SSM cathode maintained a relatively stable and higher H_2_ percentage (73 ± 6%) compared to CB and BP, which showed a minimal H_2_ portion in the produced gas (2 ± 3% and 2 ± 2%, respectively). The OMW-1 catalyst exhibited no H_2_ production at 600 mV, while the OMW-2 catalyst produced H_2_ only on a single day, reaching just 3%.

Overall, the higher cell voltage of 800 mV promoted H_2_ production, resulting in a higher and more stable share of H_2_ in the produced gas compared to 600 mV. This was likely due to the greater energetic gain for anodic bacteria, which could have led to increased biomass production, faster acetate degradation, and more efficient electron transfer to the anode. Additionally, the hydrogen evolution reaction requires higher theoretical applied voltage compared to cathodic methanogenesis. When considering the cathodic materials, the value of OMW-2 was comparable to that of the commercial carbon-based materials in terms of H_2_:CH_4_ ratio and cumulative production, while that of OMW-1 was suboptimal.

It must be further noted that microbial inoculants used in single-chamber MECs will vary depending on the waste or wastewater source used. This will in turn significantly influence H_2_ production, mainly due to three components: (i) microbial community (structure, metabolic capabilities, and adaptability to environmental conditions); (ii) environmental conditions (pH, temperature, salinity, and conductivity); (iii) waste/wastewater complexity (inhibitory compounds, variable organics content, oxygen). Therefore, further studies are needed for source screening and microbial monitoring to assess the universality of the findings in terms of performance under multiple conditions.

Additionally, although this study focuses on short-term operations, long-term operations need to be further explored, as performance results could be affected over time by cathode poisoning related to electrode corrosion and deterioration (e.g., erosion of the carbon structure and biochar detachment, reducing surface area and causing change in conductivity, pH, and catalytic activity), biofouling (e.g., excessive microbial growth or extracellular polymeric substances accumulation, for example, exopolysaccharides, that can lead to an increase in electrode overpotentials due to activation, bacterial metabolism, and concentration losses), inorganic salt precipitation such as carbonates, and shifts of the microbial community.^51^

### 3.2 Electrochemical Analysis

Analysis of the anode working potentials reported in Table 2 suggests that this parameter played a key role in enhancing H_2_ productivity and inhibiting CH_4_ production, particularly within the CB and SSM cathodes. At a cell voltage of 600 mV, lower H_2_ and higher CH_4_ production were observed along with more negative anode working potentials compared to 800 mV, i.e., −459 ± 22 and −322 ± 50 mV, for CB and SSM, respectively. In contrast, at 800 mV, H_2_ production was higher, and the anode potentials were less negative compared to 600 mV: −376 ± 66 mV for CB and −109 ± 85 mV for SSM. An increase in positive anode redox potentials has been correlated with greater energy gain for bacteria, faster anodic biofilm development, increased biomass accumulation, and quicker system startup (shorter lag phase).^52^ Nam et al.^53^ reported an increase in H_2_ production rates but a decrease in CH_4_ production when the anode polarization was increased. The highest H_2_ productivity, corresponding to 7.9 ± 0.3 m^3^ m^–3^ d^–1^, was observed at an anode potential of 0.2 V vs Ag/AgCl, whereas reduced H_2_ productivities were recorded when the anode was polarized at 0 V, −0.2 V, and −0.6 V vs Ag/AgCl with values of 6.9 ± 0.8, 3.6 ± 0.6, and 0.3 ± 0.1 m^3^ m^–3^ d^–1^, respectively. Furthermore, CH_4_ content decreased from 27 ± 6% to 3 ± 0% in the produced gas as the anode potential increased from −0.4 to 0.2 V vs Ag/AgCl.^53^

Cyclic voltammograms (CVs) recorded after the 800 and 600 mV polarization experiments (Figure 3) revealed significant differences in current density peaks and CV area between SSM and the other electrodes. Notably, the carbon-based electrodes (CB, BP, OMW-1, and OMW-2) exhibited a wider CV area compared to the SSM cathode, attributed to higher capacitive currents and increased capacitance due to differences in surface chemistry and morphology, such as the presence of oxygen functional groups.^21,54,55^

**Figure 3:**
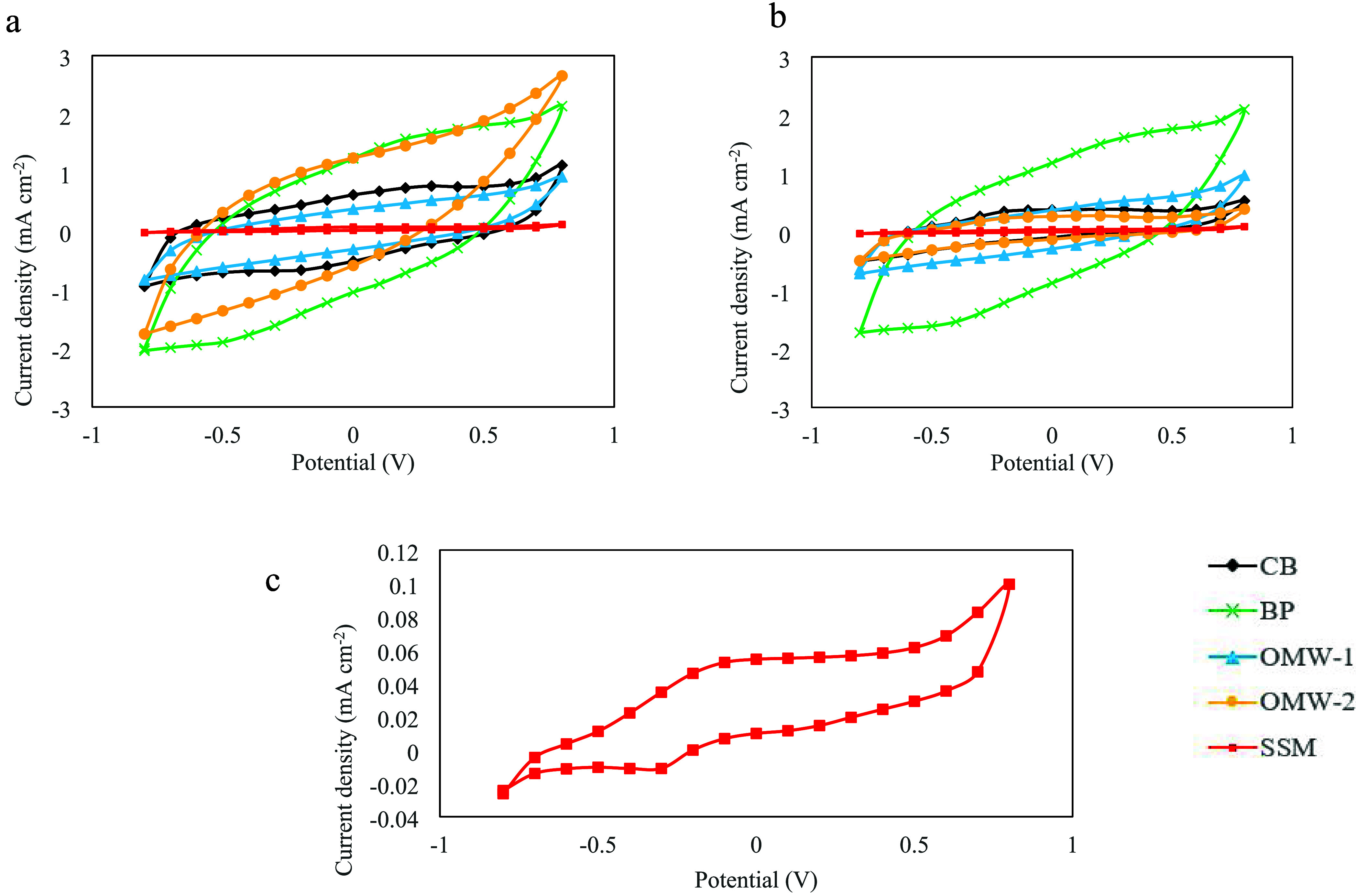
Cyclic voltammetry analysis of the cathodes after the polarization at (a) −800 and (b) −600 mV. (c) Cyclic voltammetry detail of the SSM cathode at 800 mV in which a reduction peak and a flattened oxidation peak are visible.

Focusing on the CV of the SSM cathode (Figure 3c), a flattened oxidation peak appeared around 0 V vs Ag/AgCl (0.06 mA cm^–2^), while a reduction peak was observed at −0.3 V vs Ag/AgCl (−0.01 mA cm^–2^). These peaks can be attributed to the redox activity of Fe^2+^/Fe^3+^ species present in stainless steel,^56^ the possible adsorption/desorption of hydrogen on the electrode surface,^57^ or the H_2_ redox reaction.^58^ Microbial activity on the SSM cathode was ruled out based on 16S rRNA analysis (Section 3.3). Therefore, H_2_ production on this electrode occurred solely through abiotic catalysis. Conversely, the higher current density peaks and capacitive currents observed in carbon-based cathodes suggest potential redox activity associated with electroactive microorganisms colonizing the electrode surface.^55,59,60^

### 3.3 Community Characterization

To understand the differences in H_2_ production among cathode materials, microbial community composition was interpreted by considering known metabolic roles in H_2_ evolution, H_2_ consumption, and methanogenesis.

After the acclimation period, MEC electrolyte samples (inoculum) showed a different set of most abundant genera when compared to the samples collected at the end of the 600 mV and 800 mV steps, with the majority previously found in biodegradation studies as degraders of phenol, ethanol, hydrocarbon, and aromatics (e.g., *Limnobacter*, *Advenella*, *Parvibaculum*)^61–63^ or in methanogenic granular sludge (*Fluviicola* and *Lentimicrobium*)^64^ (Figure 4). The anodic bristle (inoculum) main genera found were *Geovibrio*, *Pseudomonas*, and *Thiopseudomonas*, often present in the anodic biofilm of MFCs and MECs operated for electricity generation/H_2_ production.^65^

**Figure 4:**
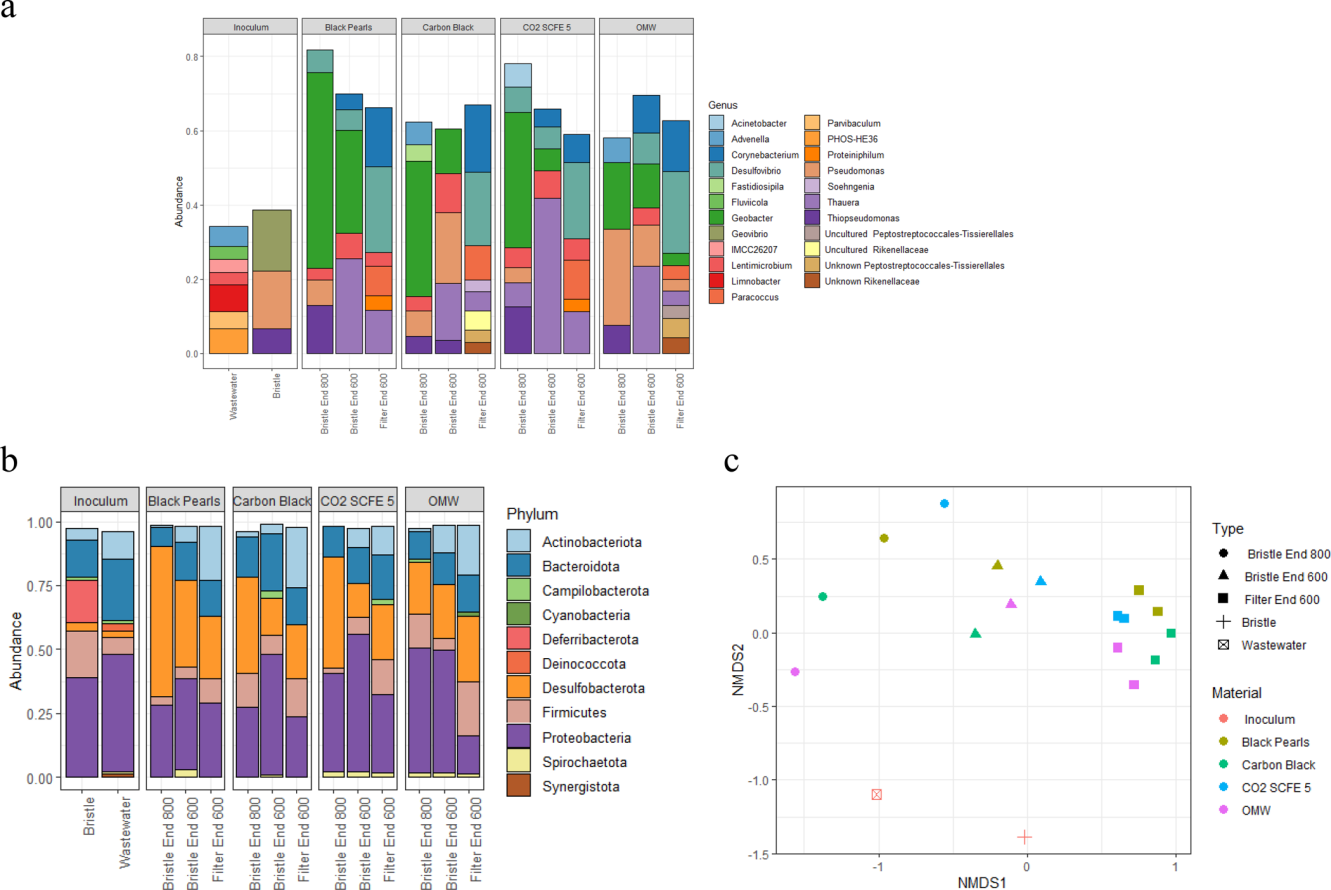
(a) Bacterial communities’ composition at the genus level at a >3% cutoff and (b) the phylum level at a >1% cutoff; relative abundances of the average values of three replicas are shown for the cathodes after the 600 mV polarization. (c) Nonmetric multidimensional scaling (NMDS).

Due to the low DNA concentration obtained from the extraction, likely caused by the limited biomass growth on the cathode, sequencing of the SSM samples was not feasible. This suggests a minimal rate or even the absence of biocatalysis, indicating that the primary H_2_ generation mechanism may be driven by abiotic catalysis. Conversely, the biocompatible surface chemistry of BP, CB, and OMW cathodes promoted the establishment of electroactive microorganisms.

Samples from the anodic bristle at the end of the 800 mV step showed some common genera with the postacclimation anodic bristle (Figure 4). *Geobacter* was the predominant exoelectrogenic bacteria owing to its extracellular electron transfer capacity to insoluble electron acceptors. *Geobacter* has been frequently found in the anodic biofilms of MFCs fed with various carbon sources.^66^ The presence of *Geobacter* may have promoted electron flux toward microbial metabolism, either through direct cathodic uptake or via direct interspecies electron transfer (DIET) with methanogens, thereby reducing the electron availability for abiotic H_2_ evolution. Additionally, *Pseudomonas* showed an involvement in electrical current generation within MFC with a higher adaptability to various environmental conditions and broader spectrum of substrates than *Geobacter*.^67^ Other less abundant genera but seemingly not linked to current generation were *Desulfovibrio*, *Advenella*, *Acinetobacter*, and *Lentimicrobium.*

Samples from the anodic bristle at the end of the 600 mV step showed the presence of *Desulfovibrio*, *Geobacter*, *Lentimicrobium*, and *Pseudomonas* as main electrogenic genera, similar to the 800 mV step (Figure 4). Additionally, *Corynebacterium* was found as the electrogenic genus.^68,69^ At 600 mV, *Geobacter* showed a reduced abundance compared to that at 800 mV, *Lentimicrobium* showed an increase, and *Desulfovibrio* and *Pseudomonas* remained similar.

Cathodic samples at the end of the 600 mV voltage application showed a similar composition in electrogenic genera in the presence of *Corynebacterium* and *Desulfovibrio* at a higher abundance than in the anodic samples, while other electrogenic genera detected at lower abundances included *Geobacter*, *Lentimicrobium*, and *Pseudomonas* (Figure 4). Under specific conditions, some *Geobacter* species can in fact thrive in cathodic biofilms, giving rise to reductive currents.^70^ Furthermore, direct electron transfer has been observed between *Geobacter* and methanogens, which can cooperate for power production.^71^ On the other hand, *Desulfovibrio* is a sulfate-reducing genus that can use molecular hydrogen as a sole energy source.^72^ Aulenta et al.^58^ reported that this genus can produce H_2_, too, but at lower cathode potential compared to the one used in this study, i.e., −0.9 V vs SHE, while no H_2_ production was observed at −0.5 and −0.7 V vs SHE. In the present study, *Desulfovibrio* was therefore more likely to act as a hydrogen sink rather than a hydrogen producer, thereby directly contributing to the lower net H_2_ recovery observed.

Additionally, *Paracoccus* and *Proteiniphilum* have emerged as new genera. *Paracoccus* species can grow autotrophically, heterotrophically, and mixotrophically; when growing autotrophically at the cathode, it can use electrons directly (electroautotrophy).^73^ It is further reported that *Paracoccus* is able to transfer electrons to other bacteria by means of a peculiar structure with the DIET mechanism.^74^ *Paracoccus* has also been recently studied and reported as hydrogen oxidizing bacteria (HOB), thus able to use H_2_ as an energy source, for single cell protein (SCP) production.^59,75^ The emergence of *Paracoccus* suggests an alternative electron sink through electroautotrophy and hydrogen oxidation, diverting cathodic electrons away from abiotic proton reduction and thus decreasing measurable H_2_ accumulation. *Proteiniphilum* is a fermentative acetogen that can use CO_2_ and H_2_ as carbon and energy sources, respectively.^59,76^

The presence of electrotrophic microorganisms and HOB could explain the lower production of H_2_ using the carbon-based cathode when compared to SSM. Being highly biocompatible and offering a multitude of functional groups for attachment and electron transfer,^77,78^ these carbon-based cathodes seemed to have fostered microbial growth and electroactive biofilm formation, as proved by CV, too, but unfortunately also toward H_2_ consuming bacteria that could have used the desired product (i.e., H_2_) for the synthesis of other compounds such as organic acids or simply biomass.

Overall, when looking at the beta-diversity, the communities at 600 mV (for both filters and bristles, separated) show a similar composition, which possibly also contributes to the similar results across the different electrodes’ materials (Table 2, Figure 2). On the other hand, the bristles’ communities at 800 mV show a more varied composition across electrode materials. The lower H_2_ production in OMW-1 could be possibly linked to the trend of lower abundance of *Desulfovibrio*, *Geobacter*, and *Lentimicrobium*. Still, the presence of other key players that determine a different H_2_ production cannot be excluded at abundances below 3%.

Hydrogen gas can be produced effectively by anaerobic organisms under low oxygen concentration through a multitude of pathways (e.g., fermentation, electroreduction of H^+^), and due to the diversity of genes and bacterial species linked to hydrogen production, high production yields can be achieved with the selection of appropriate substrates and media supporting hydrogenases activities.^79^ Bacteria, which have the potential to have a leading role in H_*2*_ production, have been identified in the genera *Clostridium*, *Enterobacter*, *Klebsiella*, *Citrobacter*, and *Bacillus*;^64,79^ however, multiple genera and species have been used in H_2_-production studies.^80^ These genera accounted for a small portion of the communities of these MECs (<1%) and mainly at the anodes, indicating that possibly other genera are contributing to H_2_ production (Figure S1). After the polarization at 800 mV, the absence of these genera in the OMW-1 sample concords however with the H_2_ production data which for this sample dropped to 0%. After the polarization at 600 mV, there is a general trend of reduction of these genera, which, coupled with the higher CH_4_ production, could have led to an almost absent H_2_ production.

Ivikodak was used to predict the variation in enzyme abundance associated with methanogenesis based on 16S rRNA sequences (Figure S2). Methanogenesis is undesirable within MECs as it results in substrate competition between methanogens and electroactive bacteria, leading to a reduction in electricity production and electron or H_2_ consumption at the cathode.^81^ Although methanogenesis is carried out mainly by methanogenic Archaea, abundant bacterial enzymes (EC 6.2.1.1 - acetyl-CoA synthetase, EC 2.3.1.8 phosphate acetyltransferase, and EC 2.7.2.1 - acetate kinase) were retrieved within the CH_4_ metabolism pathway (Kegg map: ec00680), which are involved in the metabolism of acetate, which can impact substrate availability for methane production from acetate. Bristles collected from the anode of the OMW-1 MECs at the end of the 800 mV polarization experiment showed a trend of highest potential activity of acetate metabolism and possibly CH_4_ production, which is in accordance with the CH_4_ production data that indicates the highest production, at this step, for the OMW-1 MECs among all materials. At the end of the 600 mV polarization, the cathode of the OMW-2 MECs followed by anodic samples of the CB and BP MECs showed the highest acetate metabolism activity.^82^ Further details of the microbiological characterization can be found in the Supporting Information.

Overall, these results indicate that the lower hydrogen yields were not due to limited electrochemical activity but rather to the establishment of a metabolically diverse biofilm. In contrast, SSM cathodes favored abiotic hydrogen evolution due to limited microbial colonization.

### 3.4 H_2_ and CH_4_ Mixed Stream Potential

In this study, increased H_2_ production was observed for all tested materials in MEC polarized at 800 mV compared to 600 mV. The highest H_2_ STYs were achieved with cathodes that had a low surface area (SSM and CB). Thus, most of the H_2_ was likely obtained through abiotic catalysis (especially for SSM) since high values of surface area are required for a fast and efficient cathodic biofilm formation and for the promotion of electron transfer from cathode to microorganisms that are crucial for biotic H_2_ production.^83^ These results confirmed that to increase the selectivity toward H_2_ production, higher cell voltages in combination with lower surface area electrodes should be preferred. However, as observed in this study, the high surface area of the electrodes can lead to an enrichment in hydrogen-consuming microorganisms that can result in H_2_ depletion and CH_4_ production. This was particularly evident when comparing SSM and CB at 600 mV. SSM had a surface area ∼150,000 times lower than CB, which resulted in an almost complete absence of microorganisms on the surface of the SSM cathode and in negligible CH_4_ production (8 ± 2 mL L^–1^ d^–1^), while on the contrary, higher CH_4_ production was observed for CB (193 ± 67 mL L^–1^ d^–1^). Thus, in the MEC system for H_2_ production, it is crucial to steer the cathodic microbial community toward H_2_-producing bacteria instead of H_2_-oxidizing bacteria and methanogenic Archaea or to operate cathodes under abiotic conditions.

Cell voltage played a key role toward the selection of the produced gas. At 600 mV, a decrease of H_2_ was observed when compared to 800 mV. The low voltage (600 mV) probably was not optimal to reach a proper and stable cathodic polarization required to sustain H_2_ production (i.e., −0.41 V vs SHE).^9^

It should be noted that most of the MEC studies are focused on H_2_ production considering CH_4_ as a byproduct. Steering these systems toward the production of a gas mixture of H_2_ and CH_4_ can be advantageous since it gives the possibility to directly use the mixture within natural gas networks, as the allowed mix includes an abundance of maximum 10% for H_2_.^84^ The presence of H_2_ in green CH_4_ offers advantages such as increased flammability, accelerated combustion, and a lower ignition temperature compared to pure CH_4_.^85^ Furthermore, a controlled H_2_–CH_4_ mixture (10–30% H_2_) could be leveraged as a viable energy storage solution known as biohythane.^85^

For the abovementioned reasons, considering the produced H_2_–CH_4_ gas mixture obtained in this study at 600 mV, the energy productivity value of OMW-2 (i.e., 3.0 ± 0.2 kWh L^–1^ d^–1^) was statistically indistinguishable (*p* > 0.05, two-tailed *t* test) with the maximum values found with commercial biochar-based cathodes (3.3 ± 1.1 kWh L^–1^ d^–1^ with CB; 3.3 ± 1.6 kWh L^–1^ d^–1^ with BP), highlighting the promising performance of noncommercial biochar-based cathode producing the gas mixture. The higher energy productivities achieved at 600 mV when compared to 800 mV, and particularly with carbon-based cathodes (Table 2), were attributed to the higher CH_4_ production at 600 mV and to the higher energy content of CH_4_ compared to H_2_ (221 × 10^–3^ kWh mol^–1^ vs 67 × 10^–3^ kWh mol^–1^). The only two exceptions were OMW-1 and SSM. The former recorded similar values at both tested cell voltages, while the latter showed an increase at 800 mV, reaching 1.3 ± 0.0 kWh L^–1^ d^–1^, almost 1.5-times higher compared to the one observed at 600 mV, which was the lowest energy productivity observed (0.5 ± 0.0 kWh L^–1^ d^–1^). These findings highlight the importance of optimizing the CH_4_–H_2_ gas mixture, especially when biochar-based cathodes are used, to maximize energy output.

The energy yield is defined as the ratio between the energy content of the produced gas (hydrogen + methane) and the electrical energy supplied to the system. To provide a more complete and comparable evaluation of the MEC performance, Coulombic efficiency (CE) was calculated the cathodic for all electrodes tested at both 800 mV and 600 mV (Table 2). The CE quantifies the fraction of electrons derived from substrate oxidation that was effectively used for hydrogen production. During the 17 day experimental period, the synthetic medium was replaced every two days and supplemented with 2 g L^–1^ sodium acetate per cycle, for a total of 225 mg added and approximately 96 ± 1% consumed. At 800 mV, the highest CE values (Table 2) were observed for CB and SSM with 69 ± 8% and 69 ± 1%, respectively, indicating better utilization of the available electrons for hydrogen production. These values were similar to the one obtained by Son et al. using the same MEC configuration with a carbon fiber brush anode and a stainless-steel mesh cathode with a low-performance anode after 67 h. A similar value was obtained using a high-performance anode after 18 h, i.e., 70.6 ± 3.9%, while increasing the experiment duration (24 h) CE value of 113.4 ± 9.8% were reached.^48^ The biochar-based cathodes showed lower CE values, suggesting that a significant portion of electrons was lost to side reactions, such as microbial growth, methanogenesis, or hydrogen oxidation. The better performance of OMW-2 compared to OMW-1 at 800 mV was confirmed by the CE values. At 600 mV, all CE values dropped substantially. Most carbon-based electrodes produced negligible hydrogen with CE values close to zero. Only SSM retained a moderate CE (24 ± 2%), confirming that lower applied voltages were insufficient to drive effective hydrogen evolution, likely due to increased overpotentials and competing electron-consuming pathways. These findings highlight the importance of selecting appropriate cathode materials and operating voltage controls to enhance the hydrogen recovery and overall energy efficiency in MECs. Higher voltages (≥800 mV) and conductive, catalytically active materials such as stainless steel or carbon black appear more favorable for promoting selective H_2_ generation. As a final remark, although the OMW-based cathode exhibited lower H_2_ production compared to metal and commercial carbon-based electrodes, it aligns with circular economy principles by contributing to waste reduction and greenhouse gas emission mitigation while enabling the production of a H_2_/CH_4_ gas mixture with an energy content comparable to that obtained using SSM and BP- or CB-based cathodes.

## 4 Conclusions

Currently, MEC technology for H_2_ production remains at the laboratory scale and has yet to reach commercial viability. To compete with more established methods, such as water electrolysis and fossil-derived H_2_ production, further efforts are needed to develop scalable MEC bioreactors that minimize competing reactions at the cathode and prevent biological H_2_ consumption. Additionally, implementing circular economy strategies, such as utilizing byproduct recovery techniques, will help reduce both costs and environmental impacts associated with electrode materials.

This study demonstrated that increasing the applied cell voltage, from 600 to 800 mV, promoted H_2_ production in the microbial electrolysis cell. Although the metal-based cathode (SSM) showed superior performance in terms of H_2_ production at both tested potentials, the biochar-based cathode, especially CB, BP, and OMW-2, achieved notable energetic yields at lower voltage, i.e., 600 mV, producing H_2_:CH_4_ mixtures with high energetic content. Considering the noncommercial biochar-based electrodes, OMW-2 showed H_2_ production comparable to the other carbon-based electrodes at 800 mV with a good selectivity, i.e., negligible CH_4_ production. On the other hand, OMW-1 showed the lowest H_2_ production at both polarizations, nonetheless producing a H_2_:CH_4_ mixture with the highest energetic content at 800 mV compared to the other electrodes. Based on these findings, two main research directions should be explored in future studies. The first focuses on optimizing H_2_ production while reducing CH_4_ by tuning the main bioelectrochemical system parameters and configuration in order to push toward the abiotic and biotic H_2_ production with simultaneous control of H_2_ consuming microorganisms. The second explores the targeted production of a H_2_:CH_4_ mixture tailored to meet the gas grid and storage standards, reducing the need for downstream CH_4_ separation.

## Supplementary Material



## Notes

Additional discussion on 16S rRNA multi-amplicon sequencing, including graphs and tables. Anode and cathode open circuit potentials are included (PDF)

## Abbreviations

BES: bioelectrochemical system
CV: cyclic voltammetry
DIET: direct interspecies electron transfer
EAB: electroactive bacteria/biofilm
HOB: hydrogen oxidizing bacteria
GDL: gas diffusion layer
HER: hydrogen evolution reaction
MEC: microbial electrolysis cell
MES: microbial electrosynthesis cell
MFC: microbial fuel cell
PtG: Power-to-Gas
PtH: Power-to-Hydrogen
PtM: Power-to-Methane
PtX: Power-to-X
SCP: single cell protein
SHE: standard hydrogen electrode
STY: space-time yield

## References

[1] IPCC . Climate Change 2023: Synthesis Report. Contribution of Working Groups I, II and III to the Sixth Assessment Report of the Intergovernmental Panel on Climate Change; Arias, P. , Bustamante, M. , Elgizouli, I. , Flato, G. , Howden, M. , Méndez-Vallejo, C. , Pereira, J. J. , Pichs-Madruga, R. , Rose, S. K. , Saheb, Y. , Sánchez Rodríguez, R. , Ürge-Vorsatz, D. , Xiao, C. , Yassaa, N. , Romero, J. , Kim, J. , Haites, E. F. , Jung, Y. , Stavins, R. , Birt, A. , Ha, M. , Orendain, D. J. A. , Ignon, L. , Park, S. , Park, Y. , Reisinger, A. , Cammaramo, D. , Fischlin, A. , Fuglestvedt, J. S. , Hansen, G. , Ludden, C. , Masson-Delmotte, V. , Matthews, J. B. R. , Mintenbeck, K. , Pirani, A. , Poloczanska, E. , Leprince-Ringuet, N. , Péan, C. , Eds.; IPCC: Geneva, Switzerland, 2023. 10.59327/IPCC/AR6-9789291691647.

[2] International Energy Agency . Renewables 2024; 2024. www.iea.org.

[3] Logroño W., Kleinsteuber S., Kretzschmar J., Harnisch F., De Vrieze J., Nikolausz M. (2023). The Microbiology of Power-to-X Applications. FEMS microbiology reviews.

[4] Elalfy D. A., Gouda E., Kotb M. F., Bureš V., Sedhom B. E. (2024). Comprehensive Review of Energy Storage
Systems Technologies,
Objectives, Challenges, and Future Trends. Energy
Strategy Reviews.

[5] Sayed E. T., Olabi A. G., Alami A. H., Radwan A., Mdallal A., Rezk A., Abdelkareem M. A. (2023). Renewable
Energy and Energy Storage
Systems. Energies.

[6] Yang Y., Yao J., Wang H., Yang F., Wu Z., Zhang Z. (2022). Study on High
Hydrogen Yield for Large-Scale Hydrogen Fuel Storage and Transportation
Based on Liquid Organic Hydrogen Carrier Reactor. Fuel.

[7] International Energy Agency . Global Hydrogen Review 2024; 2024. www.iea.org.

[8] IRENA . A Quality Infrastructure Roadmap for Green Hydrogen; International Renewable Energy Agency 2024.

[9] Park S. G., Rhee C., Jadhav D. A., Jang J. H., Hwang M. H., Chae K. J. (2025). Enhanced Hydrogen Production in Microbial Electrolysis
Cells through a Magnetically Induced Electroactive Anode Biofilm. Chemical Engineering Journal.

[10] Noori M. T., Rossi R., Logan B. E., Min B. (2024). Hydrogen Production
in Microbial Electrolysis Cells with Biocathodes. Trends in Biotechnology.

[11] Schievano A., Berenguer R., Goglio A., Bocchi S., Marzorati S., Rago L., Louro R. O., Paquete C. M., Esteve-Núñez A. (2019). Electroactive
Biochar for Large-Scale Environmental Applications of Microbial Electrochemistry. ACS Sustainable Chemistry and Engineering.

[12] Huggins T., Wang H., Kearns J., Jenkins P., Ren Z. J. (2014). Biochar
as a Sustainable Electrode Material for Electricity Production in
Microbial Fuel Cells. Bioresour. Technol..

[13] Hristea G., Iordoc M., Lungulescu E. M., Bejenari I., Volf I. (2024). A Sustainable
Bio-Based Char as Emerging Electrode Material for Energy Storage Applications. Sci. Rep..

[14] Juergensen N., Weiler J. R., Knoll M. T., Gescher J., Edel M. (2025). Strategic
Improvement of Shewanella Oneidensis for Biocatalysis: Approach to
Media Refinement and Scalable Application in a Microbial Electrochemical
System. N. Biotechnol..

[15] Rousseau R., Etcheverry L., Roubaud E., Basséguy R., Délia M. L., Bergel A. (2020). Microbial Electrolysis Cell (MEC):
Strengths, Weaknesses and Research Needs from Electrochemical Engineering
Standpoint. Applied Energy.

[16] Murugaiyan J., Narayanan A., Naina Mohamed S. (2024). Biohydrogen Generation from Distillery
Effluent Using Baffled Up-Flow Microbial Electrolysis Cell. Water Environment Research.

[17] Karthikeyan R., Cheng K. Y., Selvam A., Bose A., Wong J. W. C. (2017). Bioelectrohydrogenesis
and Inhibition of Methanogenic Activity in Microbial Electrolysis
Cells - A Review. Biotechnol. Adv..

[18] Hou Y., Luo H., Liu G., Zhang R., Li J., Fu S. (2014). Improved Hydrogen
Production in the Microbial Electrolysis Cell by Inhibiting Methanogenesis
Using Ultraviolet Irradiation. Environ. Sci.
Technol..

[19] Zhang J., Bai Y., Fan Y., Hou H. (2016). Improved Bio-Hydrogen Production
from Glucose by Adding a Specific Methane Inhibitor to Microbial Electrolysis
Cells with a Double Anode Arrangement. J. Biosci.
Bioeng..

[20] Goren A. Y., Kilicaslan A. F., Dincer I., Khalvati A. (2024). Hydrogen Production
from Energetic Poplar and Waste Sludge by Electrohydrogenesis Using
Membraneless Microbial Electrolysis Cells. Renew.
Energy.

[21] Pepè
Sciarria T., de Oliveira M. A. C., Mecheri B., D’Epifanio A., Goldfarb J. L., Adani F. (2020). Metal-Free Activated Biochar as an
Oxygen Reduction Reaction Catalyst in Single Chamber Microbial Fuel
Cells. J. Power Sources.

[22] Goldfarb J. L., Buessing L., Gunn E., Lever M., Billias A., Casoliba E., Schievano A., Adani F. (2017). Novel Integrated Biorefinery
for Olive Mill Waste Management: Utilization of Secondary Waste for
Water Treatment. ACS Sustainable Chem. Eng..

[23] Goglio A., Carrara A., Elboghdady H. G. E., Cucina M., Clagnan E., Soggia G., De Nisi P., Adani F. (2025). The Performance of
Biochar Waste-Derived Electrodes in Different Bio-Electrochemical
Applications. J. Power Sources.

[24] Wang C. T., Sangeetha T., Ding D. Q., Chong W. T., Yan W. M. (2018). Implementation
of Surface Modified Carbon Cloth Electrodes with Biochar Particles
in Microbial Fuel Cells. Int. J. Green Energy.

[25] Marzorati S., Magni M., Campisi S., Ghiara G., Valtorta G. A., Gervasini A., Trasatti S. P. (2024). Development of Biochar-Based Composites
Electrodes from Pyrolysis of Coffee Silverskin: Microbial Fuel Cells
for Wastewater Treatment. Chem. Eng. Trans..

[26] Cabot Corporation . Vulcan XC72R Speciality Carbon Blacks; 2016. https://www.cabotcorp.com/search/?query=vulcan+xc72r (accessed 2025–01–10).

[27] Cabot Corporation . BLACK PEARLS 2000 Carbon Black; 2014. https://www.fetc.com.tw/upload/files/BLACK-PEARLS-2000pdf.pdf (accessed 2025–01–10).

[28] Pérez-Rodríguez S., Pastor E., Lázaro M. J. (2018). Electrochemical Behavior of the Carbon
Black Vulcan XC-72R: Influence of the Surface Chemistry. Int. J. Hydrogen Energy.

[29] Cangül B., Zhang L. C., Aindow M., Erkey C. (2009). Preparation of Carbon
Black Supported Pd, Pt and Pd–Pt Nanoparticles Using Supercritical
CO2 Deposition. J. Supercrit. Fluids.

[30] Lu L., Xing D., Xie T., Ren N., Logan B. E. (2010). Hydrogen
Production from Proteins via Electrohydrogenesis in Microbial Electrolysis
Cells. Biosens. Bioelectron..

[31] Gautam R., Nayak J. K., Ress N. V., Steinberger-Wilckens R., Ghosh U. K. (2023). Bio-Hydrogen Production through Microbial
Electrolysis
Cell: Structural Components and Influencing Factors. Chemical Engineering Journal.

[32] He K., Li W., Tang L., Li W., Lv S., Xing D. (2022). Suppressing
Methane Production to Boost High-Purity Hydrogen Production in Microbial
Electrolysis Cells. Environ. Sci. Technol..

[33] Maretto L., Deb S., Ravi S., Chiodi C., Manfredi P., Squartini A., Concheri G., Renella G., Stevanato P. (2022). Microbial
Diversity of Reconstituted, Degraded, and Agricultural Soils Assessed
by 16S RDNA Multi-Amplicon Sequencing. Front.
Environ. Sci..

[34] McMurdie P. J., Holmes S., Watson M. (2013). Phyloseq: An R Package for Reproducible
Interactive Analysis and Graphics of Microbiome Census Data. PLoS One.

[35] Oksanen, J. ; Simpons, G. L. ; Blanchet, F. Guillaume ; Kindt, R. ; Legendre, P. ; Minchin, P. R. ; O’Hara, R. B. ; Solymos, P. ; Stevens, M. H. H. ; Szoecs, E. ; Wagner, H. ; Barbour, M. ; Bedward, M. ; Bolker, B. ; Borcard, D. ; Carvalho, G. ; Chirico, M. ; De Caceres, M. ; Durand, S. ; Evangelista, H. B. A. ; FitzJohn, R. ; Friendly, M. ; Furneaux, B. ; Hannigan, G. ; Hill, M. O. ; Lathi, L. ; McGlinn, D. ; Ouelette, M.-H. ; Cunha, E. R. ; Smith, T. ; Stier, A. ; Ter Braak, C. J. F. ; Weedon, J. Package “Vegan”; 2024. https://github.com/vegandevs/vegan.

[36] Nagpal S., Haque M. M., Singh R., Mande S. S. (2019). IVikodak-A Platform
and Standard Workflow for Inferring, Analyzing, Comparing, and Visualizing
the Functional Potential of Microbial Communities. Front. Microbiol..

[37] Pawar, A. A. ; Karthic, A. ; Lee, S. ; Pandit, S. ; Jung, S. P. Microbial Electrolysis Cells for Electromethanogenesis: Materials, Configurations and Operations. Environmental Engineering Research; Korean Society of Environmental Engineers February 1, 2022. 10.4491/eer.2020.484.

[38] Roubaud E., Lacroix R., Da Silva S., Bergel A., Basséguy R., Erable B. (2018). Catalysis of the Hydrogen
Evolution Reaction by Hydrogen
Carbonate to Decrease the Voltage of Microbial Electrolysis Cell Fed
with Domestic Wastewater. Electrochim. Acta.

[39] Selembo P. A., Merrill M. D., Logan B. E. (2009). The Use
of Stainless Steel and Nickel
Alloys as Low-Cost Cathodes in Microbial Electrolysis Cells. J. Power Sources.

[40] Son S., Koo B., Chai H., Tran H. V. H., Pandit S., Jung S. P. (2021). Comparison
of Hydrogen Production and System Performance in a Microbial Electrolysis
Cell Containing Cathodes Made of Non-Platinum Catalysts and Binders. Journal of Water Process Engineering.

[41] Kim K. Y., Logan B. E. (2019). Nickel Powder Blended
Activated Carbon Cathodes for
Hydrogen Production in Microbial Electrolysis Cells. Int. J. Hydrogen Energy.

[42] Zhang Y., Merrill M. D., Logan B. E. (2010). The Use
and Optimization of Stainless
Steel Mesh Cathodes in Microbial Electrolysis Cells. Int. J. Hydrogen Energy.

[43] Fujinawa K., Nagoya M., Kouzuma A., Watanabe K. (2019). Conductive Carbon Nanoparticles
Inhibit Methanogens and Stabilize Hydrogen Production in Microbial
Electrolysis Cells. Appl. Microbiol. Biotechnol..

[44] Rivera I., Bakonyi P., Buitrón G. (2017). H2 Production in Membraneless Bioelectrochemical
Cells with Optimized Architecture: The Effect of Cathode Surface Area
and Electrode Distance. Chemosphere.

[45] Harrington T. D., Babauta J. T., Davenport E. K., Renslow R. S., Beyenal H. (2015). Excess Surface
Area in Bioelectrochemical Systems Causes Ion Transport Limitations. Biotechnol. Bioeng..

[46] Xie X., Criddle C., Cui Y. (2015). Design and
Fabrication of Bioelectrodes
for Microbial Bioelectrochemical Systems. This
journal is Cite this: Energy Environ. Sci.

[47] Pantea D., Darmstadt H., Kaliaguine S., Roy C. (2003). Electrical Conductivity
of Conductive Carbon Blacks: Influence of Surface Chemistry and Topology. Appl. Surf. Sci..

[48] Son S., Koo B., Chai H., Im H., Jung S. P. (2025). Optimization of
Operation Time and Efficiency in Microbial Electrolysis Cells Based
on Anode Performance and Operation Time. Journal
of Water Process Engineering.

[49] Wang A., Liu W., Cheng S., Xing D., Zhou J., Logan B. E. (2009). Source
of Methane and Methods to Control Its Formation in Single Chamber
Microbial Electrolysis Cells. Int. J. Hydrogen
Energy.

[50] Call D., Logan B. E. (2008). Hydrogen Production
in a Single Chamber Microbial Electrolysis
Cell Lacking a Membrane. Environ. Sci. Technol..

[51] Cui W., Yin S. (2025). Microbial Electrolysis
Cells for H2 Generation by Treating Acid Mine
Drainage: Recent Advances and Emerging Trends. Fuels.

[52] Hari A. R., Katuri K. P., Logan B. E., Saikaly P. E. (2016). Set Anode Potentials
Affect the Electron Fluxes and Microbial Community Structure in Propionate-Fed
Microbial Electrolysis Cells. Scientific Reports.

[53] Nam J. Y., Tokash J. C., Logan B. E. (2011). Comparison
of Microbial Electrolysis
Cells Operated with Added Voltage or by Setting the Anode Potential. Int. J. Hydrogen Energy.

[54] Martinez-Alvarez O., Miranda-Hernandez M. (2008). Characterization
of Carbon Pastes as Matrices in Composite
Electrodes for Use in Electrochemical Capacitors. Carbon - Sci. Tech.

[55] Carrillo-Peña D., Escapa A., Hijosa-Valsero M., Paniagua-García A. I., Díez-Antolínez R., Mateos R. (2024). Bioelectrochemical
Enhancement of Methane Production from Exhausted Vine Shoot Fermentation
Broth by Integration of MEC with Anaerobic Digestion. Biomass Convers. Biorefin..

[56] Liu S., Gu R., Diao X., Liang D., He W. (2024). Electrocatalytic
Hydrogen
Evolution and In-Situ Observation of Hydrogen Microbubbles Evolution
on Stainless Steel Meshes with Various Mesh Numbers. Int. J. Hydrogen Energy.

[57] Olivares-Ramírez J. M., Campos-Cornelio M. L., Uribe Godínez J., Borja-Arco E., Castellanos R. H. (2007). Studies on the Hydrogen Evolution Reaction on Different
Stainless Steels. Int. J. Hydrogen Energy.

[58] Aulenta F., Catapano L., Snip L., Villano M., Majone M. (2012). Linking Bacterial
Metabolism to Graphite Cathodes: Electrochemical Insights into the
H2-Producing Capability of Desulfovibrio Sp. ChemSusChem.

[59] Soggia G., Goglio A., Cristiani P., Luciani I., Clagnan E., Adani F. (2024). Bioelectrochemical Protein Production Valorising NH3-Rich Pig Manure-Derived
Wastewater and CO2 from Anaerobic Digestion. Renew. Energy.

[60] Zheng X., Xie J., Chen W., Liu M., Xie L. (2024). Boosting Anaerobic
Digestion of Long Chain Fatty Acid with Microbial Electrolysis Cell
Combining Metal Organic Framework as Cathode: Biofilm Construction
and Metabolic Pathways. Bioresour. Technol..

[61] Mori J. F., Kanaly R. A., Parales R. E. (2020). Multispecies
Diesel Fuel Biodegradation
and Niche Formation Are Ignited by Pioneer Hydrocarbon-Utilizing Proteobacteria
in a Soil Bacterial Consortium. Appl. Environ.
Microbiol..

[62] Chen Y., Feng X., He Y., Wang F. (2016). Genome Analysis of
a Limnobacter Sp. Identified in an Anaerobic Methane-Consuming Cell
Consortium. Front. Mar. Sci..

[63] Wang X., Jin D., Zhou L., Wu L., An W., Zhao L. (2014). Draft Genome
Sequence of Advenella Kashmirensis Strain W13003, a Polycyclic Aromatic
Hydrocarbon-Degrading Bacterium. Genome Announc..

[64] Sun L., Toyonaga M., Ohashi A., Tourlousse D. M., Matsuura N., Meng X. Y., Tamaki H., Hanada S., Cruz R., Yamaguchi T., Sekiguchi Y. (2016). Lentimicrobium
Saccharophilum Gen. Nov., Sp. Nov., a Strictly Anaerobic Bacterium
Representing a New Family in the Phylum Bacteroidetes, and Proposal
of Lentimicrobiaceae Fam. Nov.. Int. J. Syst.
Evol. Microbiol..

[65] Almatouq A., Babatunde A. O., Khajah M., Webster G., Alfodari M. (2020). Microbial
Community Structure of Anode Electrodes in Microbial Fuel Cells and
Microbial Electrolysis Cells. Journal of Water
Process Engineering.

[66] Kondaveeti S., Lee S. H., Park H. D., Min B. (2020). Specific Enrichment
of Different Geobacter Sp. in Anode Biofilm by Varying Interspatial
Distance of Electrodes in Air-Cathode Microbial Fuel Cell (MFC). Electrochim. Acta.

[67] Pepè
Sciarria T., Arioli S., Gargari G., Mora D., Adani F. (2019). Monitoring Microbial Communities’ Dynamics during the Start-up
of Microbial Fuel Cells by High-Throughput Screening Techniques. Biotechnology Reports.

[68] Lee S. Y., Min J., Lee S., Fitriana H. N., Kim M. S., Park G. W., Lee J. S. (2019). Bioelectricity Generation
by Corynebacterium Glutamicum
with Redox-Hydrogel-Modified Carbon Electrode. Applied Sciences.

[69] Zhao H., Kong C. H. (2018). Enhanced Removal of P-Nitrophenol in a Microbial Fuel
Cell after Long-Term Operation and the Catabolic Versatility of Its
Microbial Community. Chemical Engineering Journal.

[70] Heidary N., Kornienko N., Kalathil S., Fang X., Ly K. H., Greer H. F., Reisner E. (2020). Disparity of Cytochrome Utilization
in Anodic and Cathodic Extracellular Electron Transfer Pathways of
Geobacter Sulfurreducens Biofilms. J. Am. Chem.
Soc..

[71] Deng Q., Su C., Lu X., Chen W., Guan X., Chen S., Chen M. (2020). Performance and Functional Microbial Communities of Denitrification
Process of a Novel MFC-Granular Sludge Coupling System. Bioresour. Technol..

[72] Matias P. M., Pereira I. A. C., Soares C. M., Carrondo M. A. (2005). Sulphate Respiration
from Hydrogen in Desulfovibrio Bacteria: A Structural Biology Overview. Prog. Biophys. Mol. Biol..

[73] Perazzoli S., De Santana Neto J. P., Soares H. M. (2020). Anoxic-Biocathode Microbial Desalination
Cell as a New Approach for Wastewater Remediation and Clean Water
Production. Water Sci. Technol..

[74] Lin S., Tang W., Xiao Y., Zan F., Liu X., Chen G., Hao T. (2024). Sulfur Bacteria-Reinforced
Microbial
Electrochemical Denitrification. Bioresour.
Technol..

[75] Dou J., Huang Y., Ren H., Li Z., Cao Q., Liu X., Li D. (2019). Autotrophic,
Heterotrophic, and Mixotrophic Nitrogen
Assimilation for Single-Cell Protein Production by Two Hydrogen-Oxidizing
Bacterial Strains. Appl. Biochem. Biotechnol..

[76] Wu K. K., Zhao L., Wang Z. H., Sun Z. F., Wu J. T., Chen C., Xing D. F., Yang S. S., Wang A. J., Zhang Y. F., Ren N. Q. (2024). Simultaneous
Biogas Upgrading and
Medium-Chain Fatty Acids Production Using a Dual Membrane Biofilm
Reactor. Water Res..

[77] Hemdan B. A., El-Taweel G. E., Naha S., Goswami P. (2023). Bacterial Community
Structure of Electrogenic Biofilm Developed on Modified Graphite Anode
in Microbial Fuel Cell. Sci. Rep..

[78] Marzorati S., Goglio A., Fest-Santini S., Mombelli D., Villa F., Cristiani P., Schievano A. (2019). Air-Breathing Bio-Cathodes Based
on Electro-Active Biochar from Pyrolysis of Giant Cane Stalks. Int. J. Hydrogen Energy.

[79] Lertsriwong S., Glinwong C. (2020). Newly-Isolated Hydrogen-Producing
Bacteria and Biohydrogen
Production by Bacillus Coagulans MO11 and Clostridium Beijerinckii
CN on Molasses and Agricultural Wastewater. Int. J. Hydrogen Energy.

[80] Merugu R., Gothalwal R., Girisham S., Reddy S. M., Merugu R., Gothalwal R., Reddy · S M (2021). Bacterial Hydrogen Production: Prospects
and Challenges. BioEnergy Res.: Biomass Waste
Energy.

[81] Bagchi S., Behera M. (2021). Methanogenesis Suppression
and Increased Power Generation
in Microbial Fuel Cell during Treatment of Chloroform Containing Wastewater. Process Safety and Environmental Protection.

[82] Zhen G., Zheng S., Lu X., Zhu X., Mei J., Kobayashi T., Xu K., Li Y. Y., Zhao Y. (2018). A Comprehensive
Comparison of Five Different Carbon-Based Cathode Materials in CO2
Electromethanogenesis: Long-Term Performance, Cell-Electrode Contact
Behaviors and Extracellular Electron Transfer Pathways. Bioresour. Technol..

[83] Vassilev I., Dessì P., Puig S., Kokko M. (2022). Cathodic Biofilms –
A Prerequisite for Microbial Electrosynthesis. Bioresour. Technol..

[84] Altfeld K., Pichbeck D. (2013). Admissible Hydrogen
Concentrations in Natural Gas Systems. Gas for
Energy.

[85] Noori M. T., Min B. (2022). Fundamentals and Recent
Progress in Bioelectrochemical System-Assisted
Biohythane Production. Bioresour. Technol..

