# Supplementary material for: Tuning Hydrogen versus Methane Production on Sustainable Biochar-Based Cathodes in Microbial Electrolysis Cells by Voltage Control
Source: ACS Omega. 2026 Mar 5;11(10):16922–35. doi: 10.1021/acsomega.6c00714 (PMC13000772; doi:10.1021/acsomega.6c00714)
Supplement: Supplementary file 1 [file ao6c00714_si_001.pdf]

**Tuning hydrogen versus methane production on sustainable biochar-based cathodes in microbial electrolysis cells by voltage control**

Gabriele Soggia[a], Andrea Goglio[a], Elisa Clagnan[a,*], Tommy Pepè Sciarria[a], Barbara Mecheri[b], Alessandra D' Epifanio[b], Jillian L. Goldfarb[c], Piergiorgio Stevanato[d], Pierangela Cristiani[e], Fabrizio Adani[a]

[a] Gruppo Ricicla Lab., Department of Agricultural and Environmental Science, University of Milan, Via Celoria 2, 20133, Italy.

[b] Department of Chemical Science and Technologies, University of Rome Tor Vergata, Via della Ricerca Scientifica, 00133, Rome, Italy

[c] Smith School of Chemical and Biomolecular Engineering, Cornell University, Ithaca, NY, 14853, USA

[d] Department of Agronomy, Food, Natural Resources, Animals and Environment (DAFNAE), University of Padova, Viale dell'Università 16, 35020, Legnaro, Italy

[e] RSE-Ricerca sul Sistema Energetico S.p.A., Via Rubattino 54, 20134 Milano, Italy

**Supporting information content**

Figure S1

16S rRNA

Table S1

Figure S2

Figure S3

Figure S4

Table S2

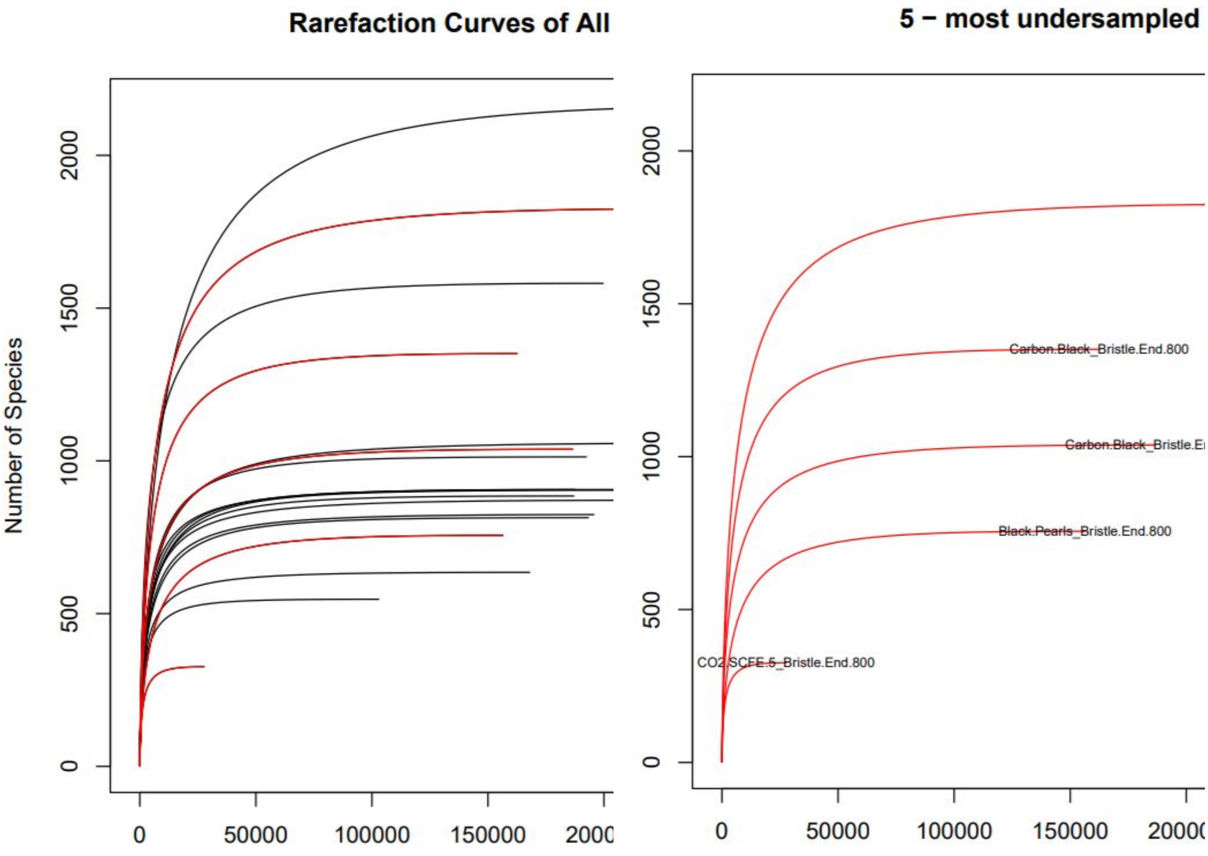


**Figure S1.** Rarefaction curves.

**16S rRNA**

The 16S sequencing produced between 35,823 and 342,139 reads with an average of 226,358 reads per sample. Bioinformatic analyses produced between 27,664 and 317,796 reads. All samples plateaued when analysing rarefaction curves (Fig. S2).

a b c

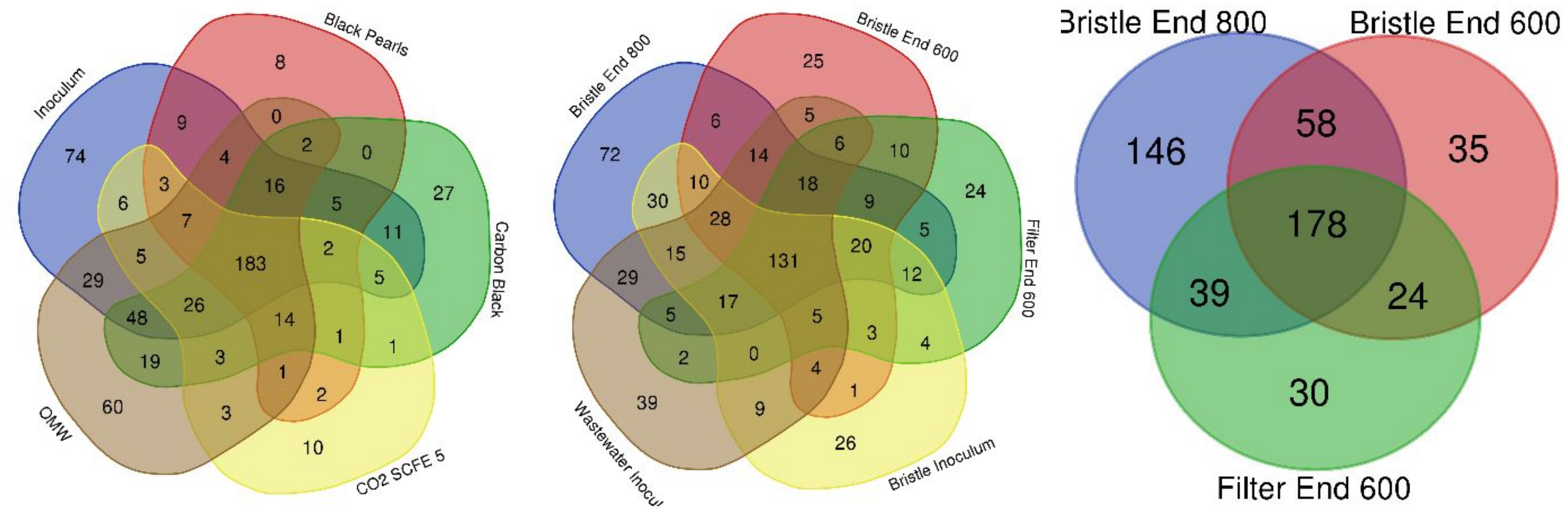


**Figure S2.** Venn diagrams showing compartmental core microbiota of genera distributions for the materials used for the filters (a), and for the type of samples (b,c).

Different materials and polarization steps showed similar observed richness and evenness. In terms of alpha-diversity all materials showed lower diversity ($p<0.05$) when compared to the inoculum while samples showed similar diversity among materials and among polarization steps (Table S1).

**Table S1**. Average anode and cathode open circuit voltage. The reported values are expressed as mean ± standard deviation (n = 3). Means followed by the same letter are not statistically different ($p < 0.05$; ANOVA, Tukey test).

| **Electrode** | **Voltage** (mV) | **Anode open circuit potential** (mV) | **Cathode open circuit potential** (mV) |
|---|---|---|---|
| OMW-1 | 600 | -452 ± 31ab | -947 ± 32ab |
| | 800 | -424 ± 34ab | -880 ± 48ab |
| OMW-2 | 600 | -452 ± 20ab | -935 ± 50ab |
| | 800 | -414 ± 30bc | -844 ± 75bc |
| BP | 600 | -444 ± 16ab | -936 ± 12ab |
| | 800 | -446 ± 27ab | -933 ± 30ab |
| CB | 600 | -459 ± 22a | -931 ± 111a |
| | 800 | -376 ± 66c | -808 ± 35c |
| SSM | 600 | -322 ± 50d | -705 ± 32d |
| | 800 | -109 ± 85e | -693 ± 54e |

At a genus level, all materials shared a total of 183 genera, the samples of the inoculum showed a higher amount of low abundant genera when compared from the other samples, concording with the results of alpha-diversity, and indicating a more diverse and probably less specialized community possibly caused by the mix of wastewater used for maintaining this culture (Figure 1, Figure S3).

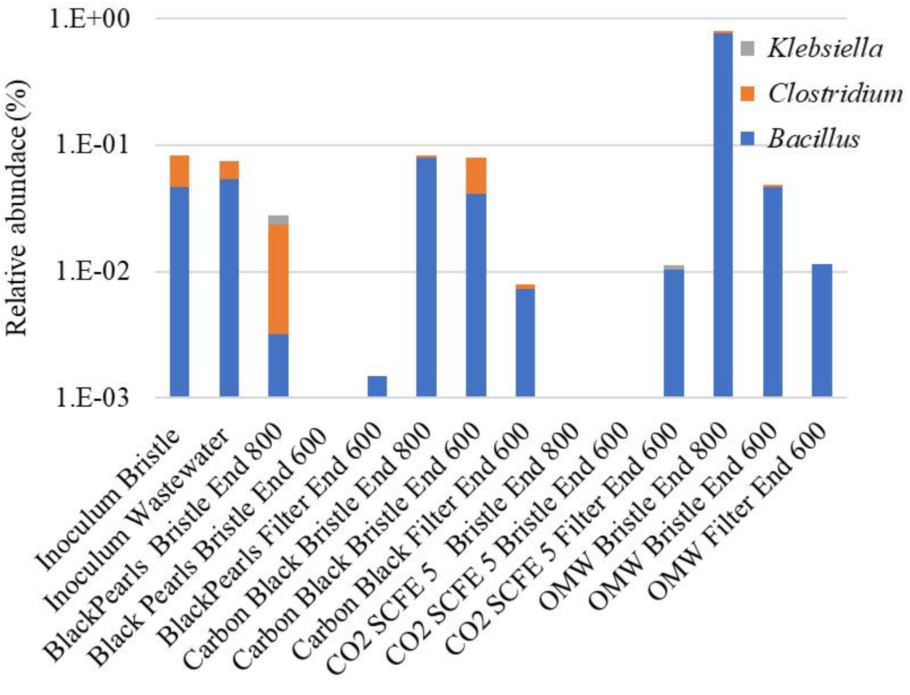


**Figure S3.** Bar plot showing the abundances of main $H_2$ producing bacteria.

From the microbial community analysis, Proteobacteria (relative abundance: 15-54%), Bacteroidota (7-24%) and Firmicutes (2-21%) phyla were present across all samples (Figure 2b). Proteobacteria, Bacteroidota and Firmicutes are commonly found in bioelectrochemical studies as Bacteroidota are fermentative generalist bacteria while Proteobacteria and Firmicutes comprehend a wide number of exoelectrogenic species (Almatouq et al., 2020; Ni et al., 2020; Ye et al., 2019). Other abundant phyla were Actinobacteria (1-24%), have been commonly found in MFCs studies (Sarranyadhevi et al., 2014), and especially on the cathodes at the end of the 600 mV polarization, Desulfobacteriota (Proteobacteria subdivision) (2-59%), across all samples but not in the inoculum (Sarranyadhevi et al., 2014).

MEC electrolyte samples further showed a different set of most abundant genera when compared to the other samples (Figure 2a). Main genera in the MEC electrolyte were previously found in biodegradation studies. *Limnobacter* (average abundance: 7%), for example, includes species with the ability of sulfur oxidization, ethanol fermentation and phenol degradation (Chen et al., 2016), *Advenella* (7%) comprehends polycyclic aromatic hydrocarbons and phenol degradaders (Li et al., 2020; Wang et al., 2014), *Parvibaculum* (5%) is a purple bacterium involved in the aromatic hydrocarbon degradation (Mori & and Kanaly, 2020) while *Fluviicola* (3%) and *Lentimicrobium* (3%) contain species isolated from methanogenic granular sludge (Sun et al., 2016). On the other hand, on the anodic bristle sampled after the acclimation period (simultaneously to the MEC electrolyte) main genera found were *Geovibrio* (17%), *Pseudomonas* (15%) and *Thiopseudomonas* (7%). These genera have been found as dominant in the anodic biofilm of MFCs and MECs operated for electricity generation/hydrogen production (Almatouq et al., 2020; Obata et al., 2020) and where also found abundant across the MEC in this study.

The MECs samples from the anodic bristle at the end of the 800 mV step showed some common genera with the post-acclimation anodic bristle. Nine main genera were encountered:

*Geobacter* (min. – max. abundances:18-53%), *Pseudomonas* (4-24%) and *Thiopseudomonas* (5-13%) were the most abundant in all samples. *Geobacter* is one of the better known exoelectrogenic bacteria due to its extracellular electron transfer capacity to insoluble electron acceptors and has been frequently found in the anodic biofilms of MFCs fed with various C-sources (Kondaveeti et al., 2020; Pepè Sciarria et al., 2019). Geobacter *sulfurreducens* is considered an electrogenic model bacterium and currently has the highest power density recorded on electrodes (Ren, 2021). Under specific conditions, some *Geobacter* species can also thrive in cathodic biofilm giving rise to reductive currents (Heidary et al., 2020). Furthermore, direct electron transfer has been observed between *Geobacter* and methanogens which can cooperate for power production (Deng et al., 2020). *Pseudomonas* showed also an involvement in electrical current generation within MFC and possess the advantage over *Geobacter* of a higher adaptability to various environmental conditions and broader spectrum of substrates (He et al., 2012; Pepè Sciarria et al., 2019; Qi et al., 2024; Yong et al., 2015) while *Thiopseudomonas* has been found in BES particularly at the anode of MFCs (Li et al., 2021; Rago et al., 2018) involved with ammonium oxidation, sulfite oxidation and nitrite reduction (Joicy et al., 2020; Tan et al., 2023, 2015). Less abundant genera were *Desulfovibrio* (0-7%), *Fastidiosipila* (0-7%), *Advenella* (0-7%), *Acinetobacter* (0-6%), *Thauera* (0-6%) and *Lentimicrobium* (1-3%). All these genera have been previously reported in MFCs studies mostly at the anodic side. *Fastidiosipila*, anaerobic/microaerobic bacteria, is the only genera that does not seem to be involved in electrogenic activities and has been previously found in digester methanogenic communities converting different compounds to acetic acid and $CO_2$ (Guan et al., 2021; Han et al., 2019). *Desulfovibrio* has been reported to be an exoelectrogen with a role in enhancing current generation by transferring electrons possibly more efficiently than *Geobacter* (Almatouq et al., 2020; Kang et al., 2014). *Advenella*, sulfide oxidizing bacteria, again has been positively correlated with changes in power density and reported as

electroactive bacterial genus (Fathima et al., 2024; Z. Yang et al., 2019) while *Acinetobacter* is also known to contribute to electricity generation and aromatic compounds degradation (Almatouq et al., 2020; Pierangeli et al., 2021). *Thauera* was observed as a denitrifier with electrogenic activity which is also able to degrade aromatic compounds (N. Yang et al., 2019) and *Lentimicrobium* has been found having important roles in complex organics degradation and it is hypothesized that it might create a consortium with other electroactive microorganisms to convert acetate into electrogenic activity (Zhang et al., 2019; Zhu et al., 2022).

Samples from the anodic bristle at the end of the 600 mV step were characterized by seven main genera. Similarly to the 800 mV step, the genera *Desulfovibrio*, *Geobacter*, *Lentimicrobium*, *Pseudomonas*, *Thauera* and *Thiopseudomonas* were encountered while the only additional genera encountered was *Corynebacterium* (0-10%). *Corynebacterium* genera include species with electrogenic activity and are again often found on the anode of MFCs (Lee et al., 2019; Zhao and Kong, 2018). At this polarization, *Geobacter* (6-28%) showed a decreasing trend compared to 800 mV MEC while *Thauera* (15-42%) and *Lentimicrobium* (5-11%) had an increase in abundance. *Desulfovibrio* (2-8%), *Pseudomonas* (1-19%) and *Thiopseudomonas* (0-4%) were mainly similar.

Cathodic samples at the end of the 600 mV voltage application showed eleven main genera. Due to the low DNA concentration obtained from the extraction, likely caused by the limited biomass growth on the cathode, sequencing of the SSM samples was not feasible. This suggests a minimal rate or even absence of biocatalysis, indicating that the primary $H_2$ generation mechanism may be driven by abiotic catalysis. Cathodes at the end of the 600 mV phase showed high relative abundance of *Corynebacterium* (8-18%) and *Desulfovibrio* (2-23%). Other genera detected at lower abundances included *Geobacter* (0-3%), *Lentimicrobium* (1-6%), *Pseudomonas* (1-3%), and *Thauera* (4-12%). *Desulfovibrio* is a sulfate-reducing genus that can use molecular hydrogen as its sole energy source (Matias et al., 2005; Steger et al.,

2002). (Aulenta et al., 2012) reported that this genus can produce $H_2$ too, but at lower cathode potential compared to the one used in this study, i.e., -0.9 V vs SHE, while no $H_2$ production was observed at -0.5 and -0.7 V vs SHE. *Paracoccus* (4-10%), *Proteiniphilum* (1-4%), *Soehngenia* (1-3%) emerged as new genera. *Paracoccus* species can grow autotrophically, heterotrophically and mixotrophically. Growing autotrophically at the cathode, this species can use directly electrons being characterized as electroautotroph (Perazzoli et al., 2020). *Paracoccus* is also reported to be able transfer electrons to other bacteria by means of peculiar structure with the direct interspecies electron transfer (DIET) mechanism (Lin et al., 2024). *Paracoccus* has also been recently studied and reported as hydrogen oxidizing bacteria (HOB), thus able to use $H_2$ as energy source, for single cell protein (SCP) production (Dou et al., 2019; Matassa et al., 2015; Soggia et al., 2024). *Proteiniphilum* is a fermentative acetogen that can use $CO_2$ and $H_2$ as carbon and energy sources, respectively (Ntagia et al., 2021; Soggia et al., 2024; Wu et al., 2024).

In light of these results, the presence of electrotrophic microorganisms and HOB could explain the lower production of $H_2$ using carbon-based cathode. Being highly biocompatible and offering a multitude of functional groups for attachment and electron transfer (Hemdan et al., 2023; Marzorati et al., 2018), those cathodes seemed to have fostered the microbial growth and electroactive biofilm formation, as confirmed by CV, but unfortunately towards hydrogen consuming bacteria that could have used the desired product, i.e., $H_2$, for the synthesis of other compounds such as organic acids or simply biomass.

NMDS and PERMANOVA were used to test for main and interactive effects. These beta-diversity analysis, indicated that both materials of the cathodes (p=0.001), type of the samples (p=0.001) and their interaction (p=0.008) have a significant effect on genera composition (Figure 2). Beta-dispersion analyses further highlighted differences among the type of samples, anodes at the end of the 800 mV polarization showed a different composition from the anode

bristles ($p<0.05$) and the cathodes ($p<0.01$) collected at the end of the 600 mV step. Significant PERMANOVAs were followed by a similarity percentage test (SIMPER) to identify specific genera that cumulatively contributed to the 50% of dissimilarity between each group. When looking at the differences between type of samples, it can be seen that differences between bristle at the end of the 800 mV polarization and at the end of the 600 mV polarization was due mainly to *Thauera* (23%, $p=0.0006$), while between bristle and cathodes at the end of the 600 mV phase mainly by *Geobacter* (11%, $p=0.02$), while between anode at the end of the 800 mV phase and cathodes at the end of the 600 mV polarization mainly by *Geobacter* (17%, $p=0.004$), *Desulfovibrio* (14%, $p=0.001$) and *Corynebacterium* (10%, $p=0.0005$).

Hydrogen gas can be produced effectively by anaerobic organisms under low oxygen concentration through a multitude of pathways (e.g. fermentation, electroreduction of $H^+$), due to the diversity of genes and bacterial species linked to hydrogen production, high production yields can be achieved with the selection of appropriate substrates and media Supplementaryhydrogenases activities (Lertsriwong and Glinwong, 2020). Bacteria, that have the potential to have a leading role in $H_2$ production, have been identified in the genera *Clostridium*, *Enterobacter*, *Klebsiella*, *Citrobacter* and *Bacillus* (Lertsriwong and Glinwong, 2020; Sun et al., 2016) however, multiple genera and species have been used in $H_2$-production studies (Merugu et al., 2021). These genera accounted for a small portion of the communities of these MEC (<1%) and mainly at the anodes, indicating that possibly other genera are contributing to $H_2$ production (Figure S4). After the polarization at 800 mV, the absence of these genera in the OMW-1 sample concord however with the $H_2$ production data which for this sample dropped to 0%. After the polarization at 600 mV, there is a general trend of reduction of these genera which, coupled with the higher $CH_4$ production, could have led to the almost absent $H_2$ production.

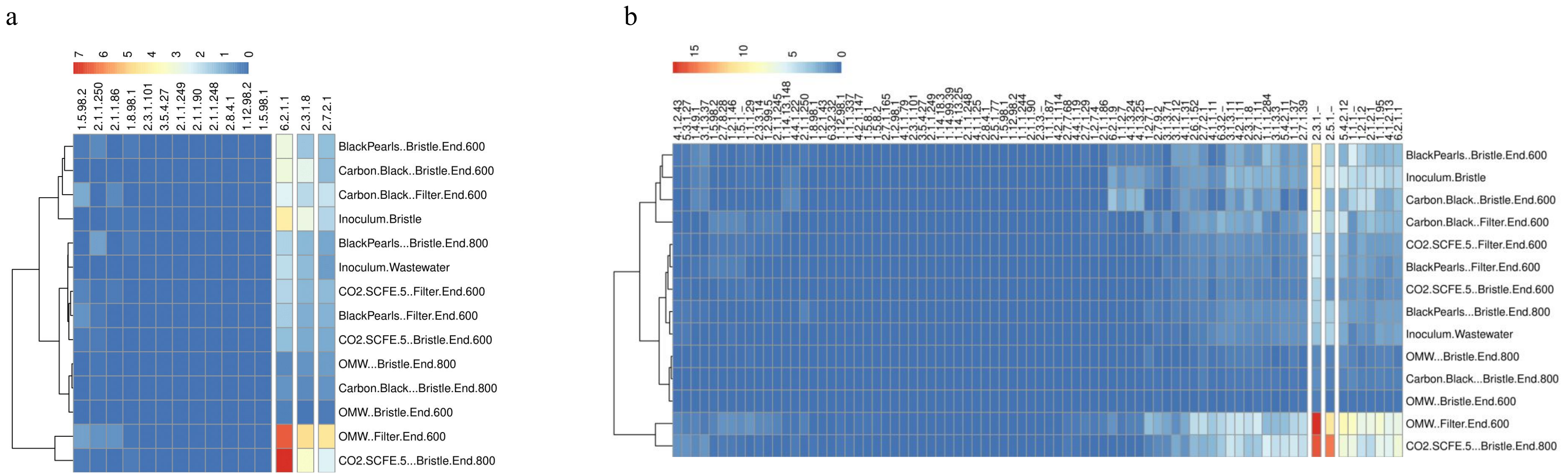


**Figure S4.** Heatmap showing the clustering of samples based on the relative percentage of iVikodak-derived bacterial community functional profile of the selected enzymes for methanogenesis (a) and methane metabolism.

Ivikodak was used to predict the variation in enzyme abundance associated with methanogenesis based on 16S rRNA sequences (Figure S5). Methanogenesis is undesirable within MECs as it results in substrate competition between methanogens and electroactive bacteria leading to a reduction in electricity production and electron or $H_2$ consumption at the cathode (Bagchi and Behera, 2021). Although methanogenesis is carried out mainly by methanogenic *Archaea*, we retrieved some abundant bacterial enzymes (EC 6.2.1.1 - acetyl-CoA synthetase, EC 2.3.1.8 phosphate acetyltransferase and EC 2.7.2.1 - acetate kinase), within the methane metabolism pathway (Kegg map: ec00680), that are involved in the metabolism of acetate which can impact the substrate availability for methane production from acetate. Bristles collected from the anode of the OMW-1 MECs at the end of the 800 mV polarization experiment showed a trend of highest potential activity of acetate metabolism and possibly methane production which is in accordance with the $CH_4$ production data that indicates highest production, at this step, for the OMW-1 MECs among all materials. At the end of the 600 mV polarization, cathode of the OMW-2 MECs followed by anodic samples of the CB and BP MECs showed highest acetate metabolism activity. Traces of known bacteria producing methane were only found in the wastewater inoculum with the genera *Rhodopseudomonas* which contain species reportedly able to carry out methanogenesis (Zheng et al., 2018).

**Table S2.** Observed richness, Shannon alpha-diversity and Pielou's evenness indexes.

| Material | Polarisation step | Observed | Shannon | Evenness |
|---|---|---|---|---|
| Inoculum | Bristle | 1826 | 5.87 | 0.78 |
| Inoculum | Wastewater | 1581 | 6.22 | 0.84 |
| Black Pearls | Bristle 600 mV | 885 | 4.79 | 0.71 |
| Black Pearls | Bristle 800 mV | 756 | 4.21 | 0.64 |
| Black Pearls | Filter 600 mV rep 1 | 547 | 4.90 | 0.78 |
| Black Pearls | Filter 600 mV rep 2 | 635 | 4.87 | 0.75 |
| Carbon Black | Bristle 600 mV | 1038 | 5.36 | 0.77 |
| Carbon Black | Bristle 800 mV | 1351 | 5.27 | 0.73 |
| Carbon Black | Filter 600 mV rep 1 | 814 | 5.09 | 0.76 |
| Carbon Black | Filter 600 mV rep 2 | 1058 | 5.29 | 0.76 |
| OMW-1 | Bristle 600 mV | 1013 | 4.88 | 0.71 |
| OMW-1 | Bristle 800 mV | 326 | 4.43 | 0.77 |
| OMW-1 | Filter 600 mV rep 1 | 906 | 5.24 | 0.77 |
| OMW-1 | Filter 600 mV rep 2 | 871 | 5.21 | 0.77 |
| OMW-2 | Bristle 600 mV | 824 | 5.09 | 0.76 |
| OMW-2 | Bristle 800 mV | 2164 | 5.43 | 0.71 |
| OMW-2 | Filter 600 mV rep 1 | 906 | 5.32 | 0.78 |
| OMW-2 | Filter 600 mV rep 2 | 904 | 5.13 | 0.75 |
